# Improved efficacy and *in vivo* cellular properties of human embryonic stem cell derivative in a preclinical model of bladder pain syndrome

**DOI:** 10.1038/s41598-017-09330-x

**Published:** 2017-08-21

**Authors:** Aram Kim, Hwan Yeul Yu, Jisun Lim, Chae-Min Ryu, Yong Hwan Kim, Jinbeom Heo, Ju-Young Han, Seungun Lee, Yoon Sung Bae, Jae Young Kim, Dong-Jun Bae, Sang-Yeob Kim, Byeong-Joo Noh, Ki-Sung Hong, Ji-Yeon Han, Sang Wook Lee, Miho Song, Hyung-Min Chung, Jun Ki Kim, Dong-Myung Shin, Myung-Soo Choo

**Affiliations:** 1 Department of Urology, Asan Medical Center, University of Ulsan College of Medicine, Seoul, 05505 Korea; 2 Department of Biomedical Sciences, Asan Medical Center, University of Ulsan College of Medicine, Seoul, 05505 Korea; 3 Department of Physiology, Asan Medical Center, University of Ulsan College of Medicine, Seoul, 05505 Korea; 4 Biomedical Engineering Center, Asan Medical Center, University of Ulsan College of Medicine, Seoul, 05505 Korea; 5 ASAN Institute for Life Sciences, Asan Medical Center, University of Ulsan College of Medicine, Seoul, 05505 Korea; 6 Department of pathology, Asan Medical Center, University of Ulsan College of Medicine, Seoul, 05505 Korea; 7 Department of Stem Cell Biology, School of Medicine, Konkuk University, Seoul, 05029 Korea; 8 Pusan National University Yangsan Hospital, Pusan National University School of Medicine, Pusan, 50612 Korea; 9 Department of Urology, Kangwon National University Hospital, Chunchon, Kangwon-do, 24289 Korea; 10 Mirae Cell Bio Co. LTD, Seoul, 05029 Korea; 11 Department of Urology, Konkuk University Medical Center, Konkuk University School of Medicine, Seoul, 05029 Korea

## Abstract

Interstitial cystitis/bladder pain syndrome (IC/BPS) is an intractable disease characterized by severe pelvic pain and urinary frequency. Mesenchymal stem cell (MSC) therapy is a promising approach to treat incurable IC/BPS. Here, we show greater therapeutic efficacy of human embryonic stem cell (hESC)-derived multipotent stem cells (M-MSCs) than adult bone-marrow (BM)-derived counterparts for treating IC/BPS and also monitor long-term safety and *in vivo* properties of transplanted M-MSCs in living animals. Controlled hESC differentiation and isolation procedures resulted in pure M-MSCs displaying typical MSC behavior. In a hydrochloric-acid instillation-induced IC/BPS animal model, a single local injection of M-MSCs ameliorated bladder symptoms of IC/BPS with superior efficacy compared to BM-derived MSCs in ameliorating bladder voiding function and histological injuries including urothelium denudation, mast-cell infiltration, tissue fibrosis, apoptosis, and visceral hypersensitivity. Little adverse outcomes such as abnormal growth, tumorigenesis, or immune-mediated transplant rejection were observed over 12-months post-injection. Intravital confocal fluorescence imaging tracked the persistence of the transplanted cells over 6-months in living animals. The infused M-MSCs differentiated into multiple cell types and gradually integrated into vascular-like structures. The present study provides the first evidence for improved therapeutic efficacy, long-term safety, and *in vivo* distribution and cellular properties of hESC derivatives in preclinical models of IC/BPS.

## Introduction

Interstitial cystitis/bladder pain syndrome (IC/BPS) is a chronic inflammatory condition of the submucosal and muscular layers of the bladder which is characterized by urothelium denudation, mast-cell activation, and sensory nerve hyperactivation^1, 2^. Many IC/BPS patients suffer from vague pelvic pain that can be exacerbated by bladder filling and is often associated with urinary frequency, urgency, and a decreased quality of life that can include sexual dysfunction, sleep dysfunction, depression, anxiety, and chronic stress^3, 4^. Although it was previously considered relatively uncommon, with a prevalence of only 0.1%, recent evidence suggests that IC/BPS may be present in >2% of females^5^. Multiple treatment strategies are used for IC/BPS including oral agents such as pentosan polysulfate^6, 7^, histamine type I receptor antagonists^8^, immunosuppressant agents^9^, monoclonal antibodies against nerve growth factor^10^, and hydrodistension of the urinary bladder and transurethral resection/coagulation of Hunner lesions^11^, but outcomes are still not satisfactory, with frequent recurrence of symptoms and Hunner lesions^12^. Therefore, treatment of IC/BPS remains a clinical challenge and further research on disease pathogenesis is required to identify curative treatments.

Recently, we reported beneficial outcomes of mesenchymal stem cells (MSCs) derived from human umbilical cord-blood (UCB) for curing IC/BPS and ketamine-induced cystitis in a rat model^13, 14^, suggesting stem cell (SC)-based therapy as a possible approach to treat IC/BPS in patients. Preclinical and clinical trial data suggest that MSCs are a practical and safe source of cells for SC-based therapies^15–19^. However, limited therapeutic efficacy and technical problems associated with large-scale *ex vivo* expansion indicate that an alternative cell source is required to obtain sufficient cell numbers of the appropriate lineage potential to treat patients with severe diseases. More importantly, direct assessment of the biological and molecular properties of engrafted cells in the pathological environment has not been performed for current MSC therapies; thus, underlying therapeutic mechanisms, tumorigenic risk after transplantation, and the optimal transplantation protocol are all unclear.

Embryonic SCs (ESCs) established from the blastocyst inner cell mass can differentiate into all cell types in our body and can be expanded *ex vivo* as immortalized cell lines^20, 21^. Based on this pluripotency and unlimited expansion potential, ESCs are considered a promising resource for regenerative medicine^22^. Recently, MSC-like cells were obtained from human ESCs (hESCs) through epithelial−mesenchymal transition by spontaneous or controlled differentiation with growth factor cocktails and supporting feeder cells (OP9), as well as a porous membrane-mediated isolation of MSCs^23, 24^. The hESCs-derived MSCs possess important advantages, including the capacity to generate a virtually unlimited supply of therapeutic cells and control differentiation to ensure optimum safety and potency before transplantation, which could in turn overcome the drawbacks of current MSC therapy. However, safety issues of hESC-based therapy must still be addressed, including the ability to form teratoma and other tumors, potential immune reactions, and the risk of differentiating into unwanted cell types.

In the present study, we demonstrate that multipotent stem cells (M-MSCs) derived from hESCs more effectively improve bladder voiding function and repair the pathological characteristics of IC/BPS than adult bone-marrow (BM)-derived cells in an IC/BPS animal model induced by instillation of hydrochloric-acid (HCl). Further, there was no evidence of any adverse outcome, such as abnormal growth, tumorigenesis, or immune-mediated transplant rejection during the 12-months of investigation. More importantly, we longitudinally monitored the *in vivo* distribution and phenotypic properties of infused M-MSCs by confocal microscopy and micro-endoscopy in living animals for 6-months after transplantation. To our knowledge, the present study provides the first evidence for the therapeutic efficacy and long-term safety, graft survival, and *in vivo* properties of hESC progeny for treating IC/BPS.

## Results

### Characterization of M-MSCs derived from hESCs

**Figure 1:**
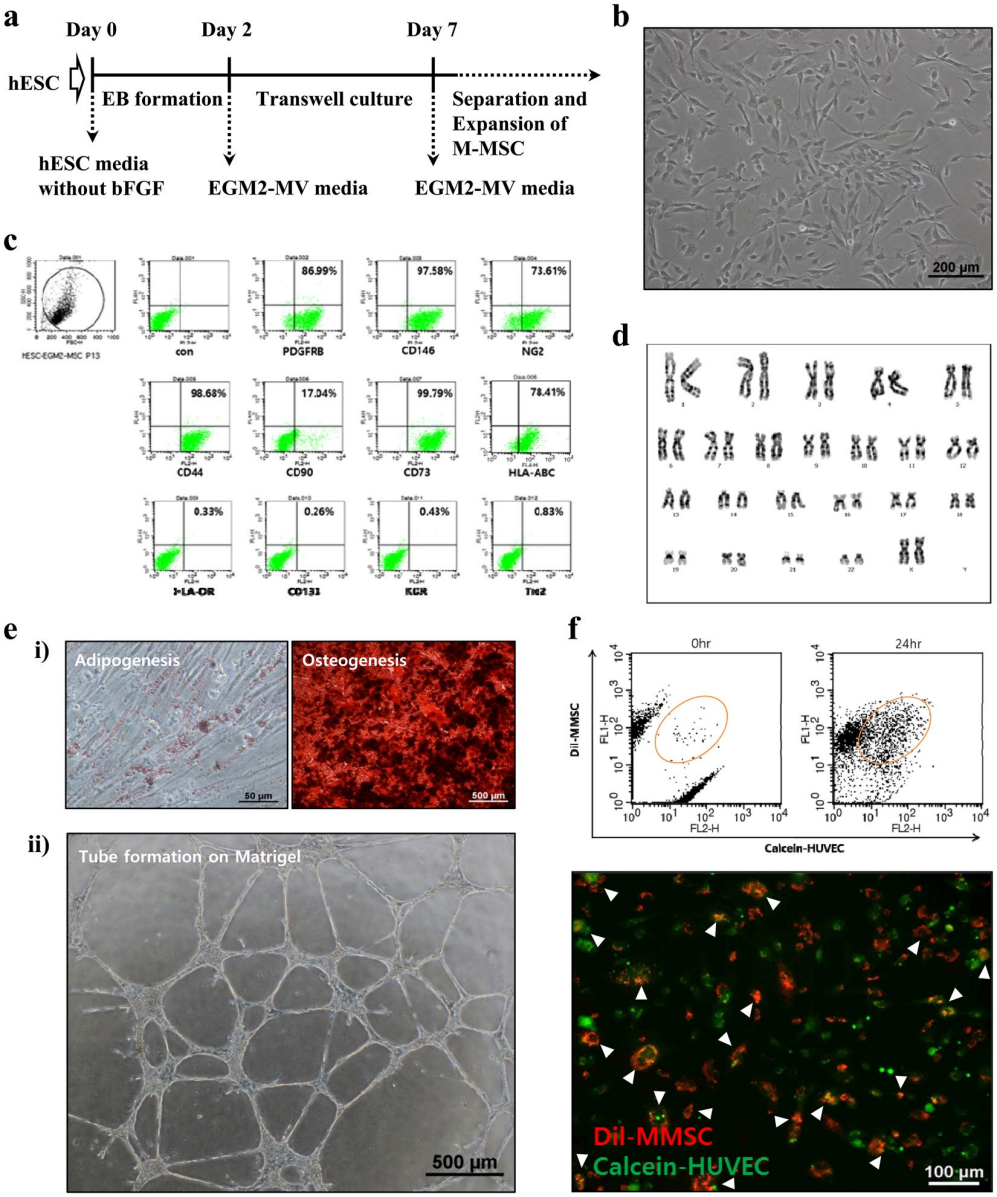
Isolation and characterization of hESC-derived M-MSCs. (**a**) Experimental scheme for the transwell-based differentiation. (**b**) Morphological characterization of M-MSCs. Scale bar = 200 μm. (**c**) M-MSCs at passage of 8 were analyzed for specific surface antigen marker expression for hMSCs (CD44, CD90, and CD73), pericytes (PDGFRB, CD146, and NG2), endothelial cells (KDR and Tie-2), hematopoietic progenitors (CD133), and MHC class I (HLA-ABC) and II (HLA-DR). (**d**) Karyotypic analysis of M-MSCs at passage 19. The isolated cells were capable of stable proliferation without chromosomal changes. (**e**) (i) Differentiation potential of M-MSCs was shown by adipogenesis (left, Oil red staining, scale bar = 50 μm) and osteogenesis (right, Alizarin red staining, scale bar = 500 μm). (ii) *In vitro* tube assembly assay. Scale bar = 500 μm. (**f**) Dye transfer (circle in the plot) increased in a coculture of DiI-labeled M-MSCs (M-MSC-DiI, red) and calcein-labeled human umbilical vein endothelial cells (HUVEC-Calcein, green). Dye-transfer between M-MSCs and HUVECs was visualized by flourescence microscopy (white arrowheads).

The hESC line H9 was differentiated by embryoid body (EB) formation for 2 days and the mesenchymal cells were isolated as those migrating to the lower compartment of a porous Transwell membrane (8 μm) over 5 days (Fig. 1a). Plating of migrated cells onto collagen-coated dishes naturally selected the CD140B^+^CD44^+^ M-MSCs, thus this controlled hESC differentiation resulted in greater than 99% pure M-MSCs within 7 days^24^. The M-MSCs exhibited several typical MSC features, including spindle and fibroblast-like morphology (Fig. 1b), expression of MSC (CD73 and CD105) and pericyte (PDGFRB, CD146, and NG2) surface antigens but little MHC class II (HLA-DR), hematopoietic progenitor (CD133), or endothelial (KDR and Tie-2) marker expression (Fig. 1c). They differentiated into mesodermal osteogenic and adipogenic lineages (Fig. 1e) and substantially downregulated markers of pluripotency (*OCT4*, *NANOG*, and *SOX2*), and upregulated expression of EMT markers such as fibronectin, consistent with MSC-like characteristics^24^. The established M-MSCs could be expanded for more than 30 passages (once per day) without identifiable chromosomal changes (Fig. 1d). Moreover, M-MSCs exhibited tube-forming capacity on Matrigel (Fig. 1e) and also formed functional junctions with blood vessel endothelial cells, as evidenced by dye-transfer assay (Fig. 1f), indicating robust pro-angiogenic potency.

### *In vivo* therapeutic potency of M-MSC for treating IC/BPS

**Figure 2:**
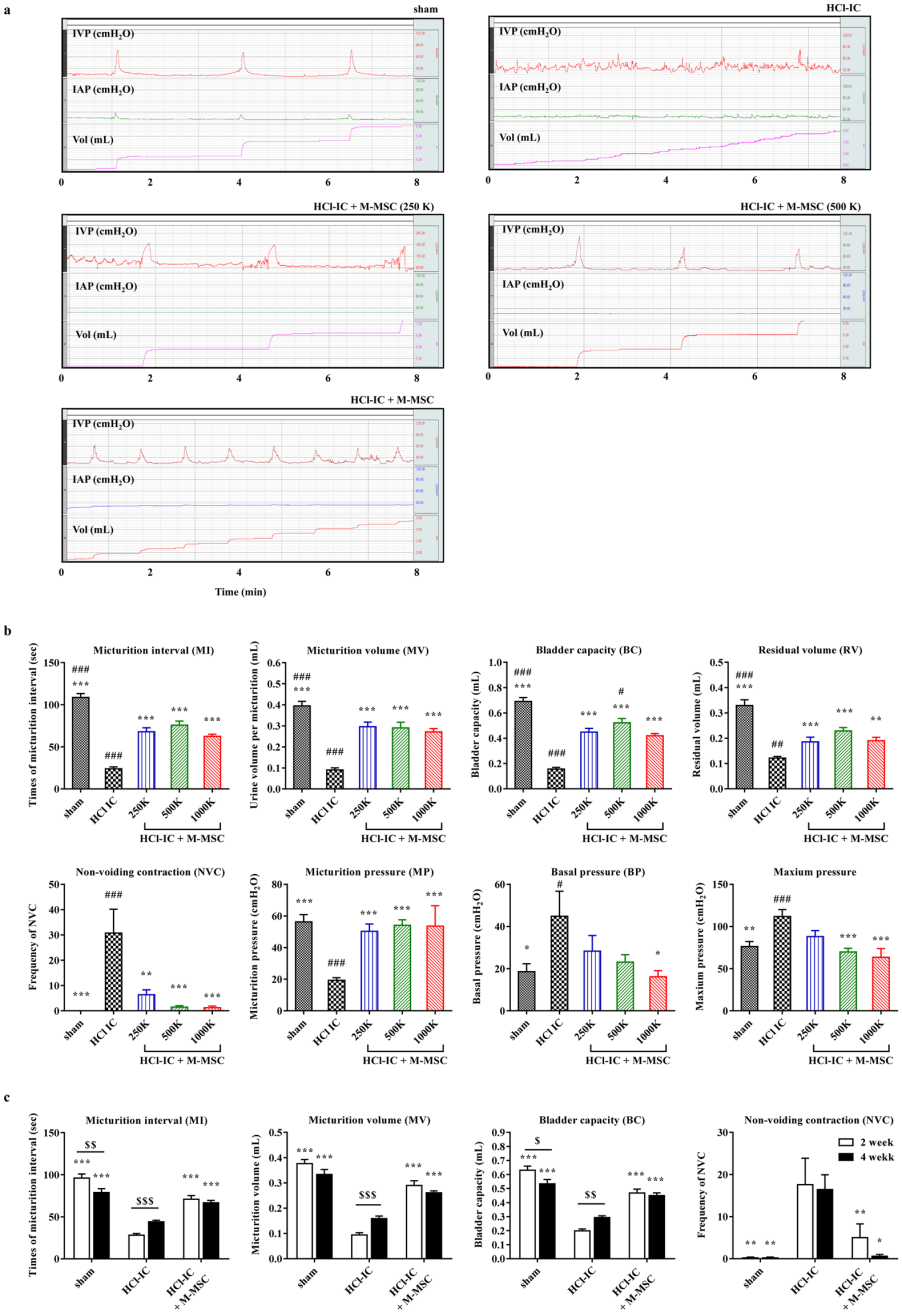
M-MSC injection restored bladder function in HCl-IC model rats. (**a**) Representative awake cystometry results and (**b**) quantitative bladder voiding parameters at 1 week after the injection of M-MSCs (8 independent animals in each group) at the indicated dosage (K = a thousand). IVP; intravesical pressure, IAP; intra-abdominal pressure. Sham: sham-operated. (**c**) Quantitative data of bladder voiding parameters at 2 or 4 weeks after the injection of 1 × 10^6^ M-MSCs (5 independent animals per group). All data are presented as the mean ± SEM, *p < 0.05, **p < 0.01, ***p < 0.001 compared to the HCl-IC group; ^#^p < 0.05, ^##^p < 0.001, ^###^p < 0.001 compared to the 1000 K group; $p < 0.05, $$p < 0.001, $$$p < 0.001 between 2 and 4 weeks group with Bonferroni post-test.

**Figure 3:**
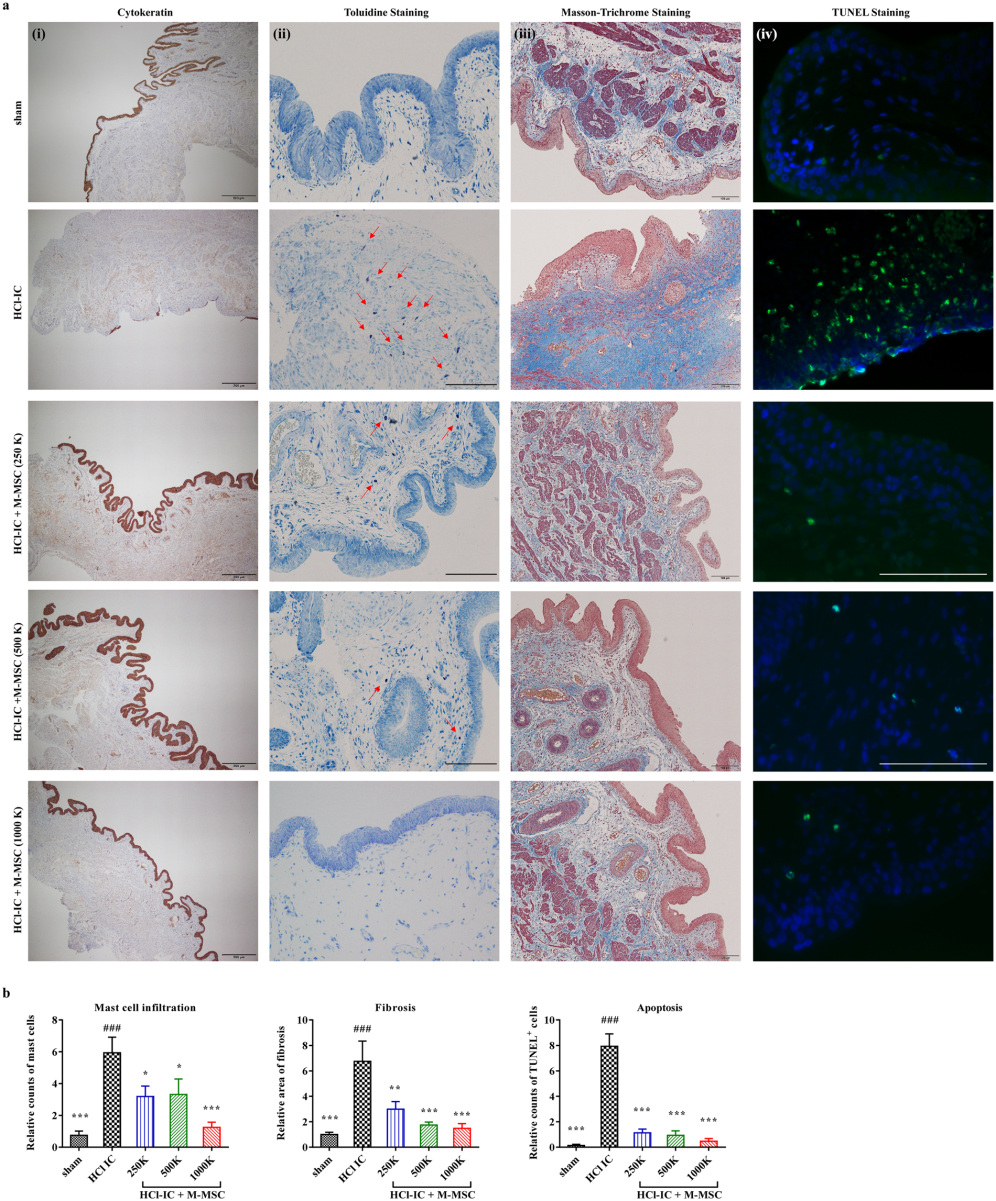
Histological analysis of M-MSC injection effects on HCl-induced bladder injury. (**a**) The immunostaining of cytokeratin (i) (magnification ×40, scale bar = 100 μm), Toluidine blue (ii) (magnification ×200, scale bar = 100 μm), Masson’s trichrome staining (iii) (magnification ×100, scale bar = 100 μm), and TUNEL assay (iv) (magnification ×400, scale bar = 100 μm) in bladder tissues of HCl-IC rats 1 week after injection of PBS vehicle or M-MSCs at the indicated dosage (K = a thousand). Arrows indicate infiltrated mast cells. Sham: sham-operated. Nuclei were stained with Mayer’s hematoxylin (i, ii, and iii) or DAPI (blue, iv). Arrows (ii) indicate infilated mast cells. (**b**) Quantification of histological examniations. Data were normalized to the sham group (n = 15). Data are presented as the mean ± SEM, *p < 0.05, **p < 0.01, ***p < 0.001 compared to the HCl-IC group; ^#^p < 0.05, ^##^p < 0.001, ^###^p < 0.001 compared to the 1000 K group with Bonferroni post-test.

We next examined the *in vivo* efficacy of M-MSCs using a IC/BPS animal model established by HCl instillation^13^. Analysis of bladder function using awake cystometry indicated that HCl instillation induced IC/BPS rats (HCl-IC group) exhibited irregular voiding and decreased micturition interval (MI) compared to sham-operated (sham) rats (23.93 ± 2.19 s vs. 108.7 ± 4.53 s; p < 0.001), as well as lower micturition volume (MV; 0.090 ± 0.010 vs. 0.395 ± 0.022 mL; p < 0.001), micturition pressure (MP; 19.36 ± 1.63 vs. 56.24 ± 4.62 cmH_2_O; p < 0.001), bladder capacity (BC; 0.156 ± 0.014 vs. 0.691 ± 0.030 mL; p < 0.001), and residual volume (RV; 0.121 ± 0.007 vs. 0.329 ± 0.022 mL; p < 0.001) (Fig. 2a and b). The IC/BPS rats also exhibited increased maximum pressure (111.90 ± 8.17 vs. 76.12 ± 5.99 cmH_2_O; p < 0.01) and bladder basal pressure (BP; 44.83 ± 11.87 vs. 18.58 ± 3.76 cmH_2_O; p < 0.05) compared to shams (Fig. 2b). A single transplantation of 1 × 10^6^ M-MSCs (HCl-IC + M-MSC group) significantly ameliorated these defective voiding parameters. This beneficial effect was observed by administration of more than 2.5 × 10^5^ M-MSCs (Fig. 2a and b) and was sustained for 2- or 4-weeks after transplantation (Fig. 2c). In particular, animals in the HCl-IC group typically exhibited increased frequency of contraction during non-voiding periods (NVC) that was significantly ameliorated by M-MSC therapy even at a low dosage (2.5 × 10^5^) of M-MSCs (Fig. 2b and c). Consistent with these functional improvements revealed by awake cystometry, administration of more than 2.5 × 10^5^ M-MSCs restored histological abnormalities in model rats, such as severe denudation of the urothelium, mast cell infiltration, fibrosis, and apoptosis (Fig. 3a,b, and Supplementary Fig. 1a), which are also characteristics of the human IC/BPS bladder^25–28^. However, little histological alteration in bladder muscles was observed in rats in all groups (Supplementary Fig. 1b).

### Effect of M-MSC on visceral hypersensitivity

**Figure 4:**
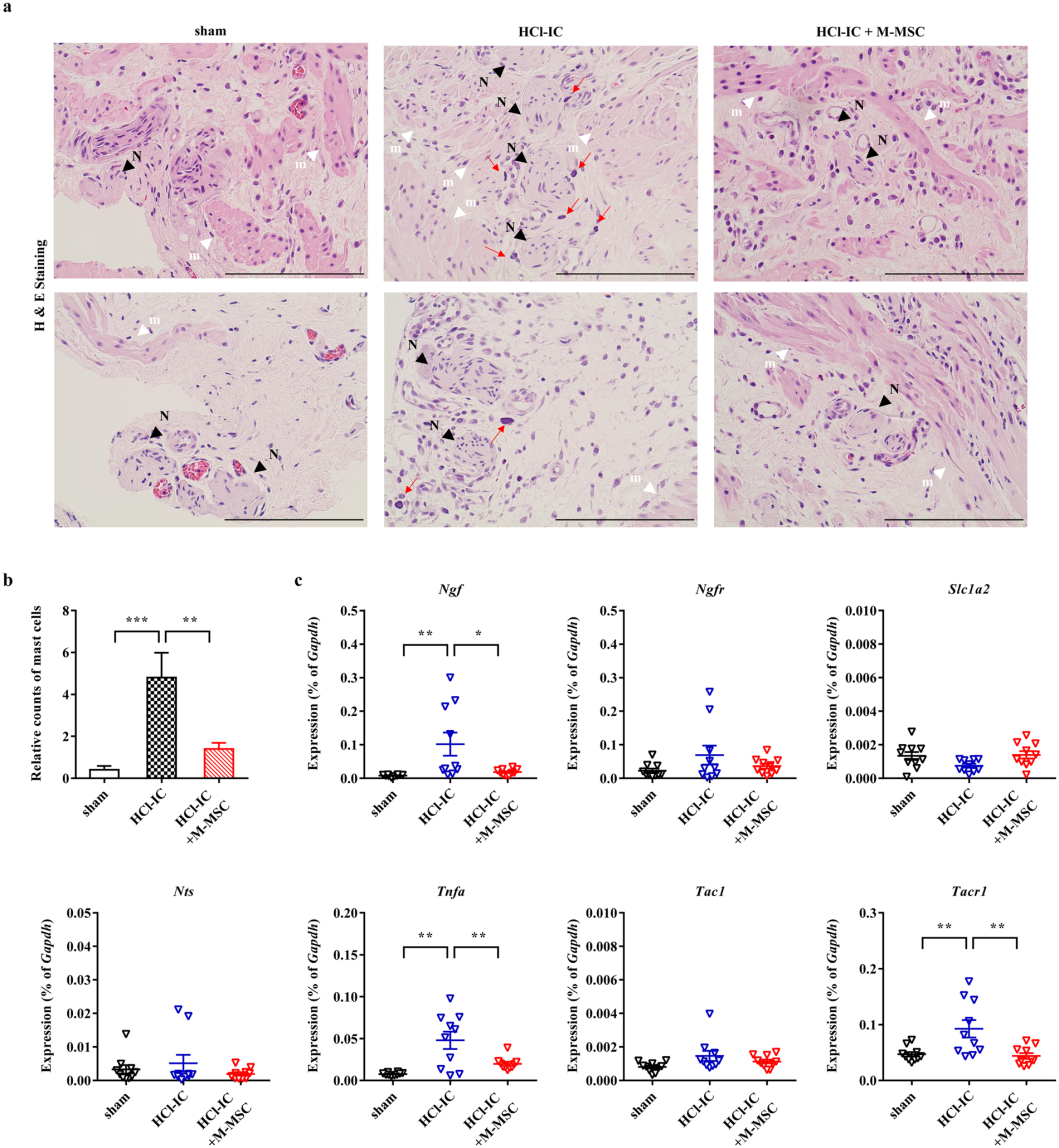
The effect of M-MSC injection on visceral hypersensitivty in HCl-IC model rats. (**a**) Hematoxylin and eosin (H & E) staining in the indicated bladder tissues (magnification ×200, scale bar = 100 μm). Nuclei were stained with Mayer’s hematoxylin. Arrows indicate the infiltrated mast cells. N (black arrowhead) and m (white arrowhead) indicate the nerve and muscle fibers, respectively. (**b**) Quantification of the mast cells infiltrated around the nerve fibers (n = 15). (**c**) RQ-PCR analyses of expression levels of *Ngf* and the genes associated with visceral hypersensitiviy in the indicated bladder tissues 1 week after the injection of 1 × 10^6^ M-MSCs or PBS vehicle into HCl-IC animals. Expression is presented as % *Gapdh* (n = 10). All data are presented as the mean ± SEM, *p < 0.05, **p < 0.01, Bonferroni post-test.

IC/BPS patients sometimes experience various other diseases involving pelvic pain, including inflammatory bowel syndrome (IBS)^29, 30^. Visceral organ communication might be a contributing variable in the overlap of symptoms in patients with IC/BPS and IBS, and increased anatomical interactions between mast cells and nerve fibers as well as an increase of nerve growth factor (NGF) after inflammation plays a pivotal role in chronic pain^31^. Thus, structural regeneration of urothelium and reduced inflammation in the bladder after M-MSC therapy might ameliorate visceral hypersensitivity in IC/BPS rats. In the histological examination, infiltrated mast cells were frequently observed near nerve fibers in the IC-HCl rat bladders; however, the anatomical interaction between mast cells and nerve fibers was significantly reduced by M-MSC therapy (Fig. 4a and b). Furthermore, gene expression analysis indicated that the bladder tissues in IC-HCl rats were characterized by the increased expression of *Ngf* and other genes associated with visceral hypersensitivity such as tumor necrosis factor-α and tachykinin receptor-1; however, the administration of M-MSCs significantly restored their induction in bladder tissues (Fig. 4c). These results suggest that M-MSC therapy could be beneficial in controlling visceral organ crosstalk as well as the severity and frequency of abdominal pain or discomfort in IC/BPS and IBS patients.

### Significance of Wnt and downstream growth factors on M-MSC therapeutic outcome

**Figure 5:**
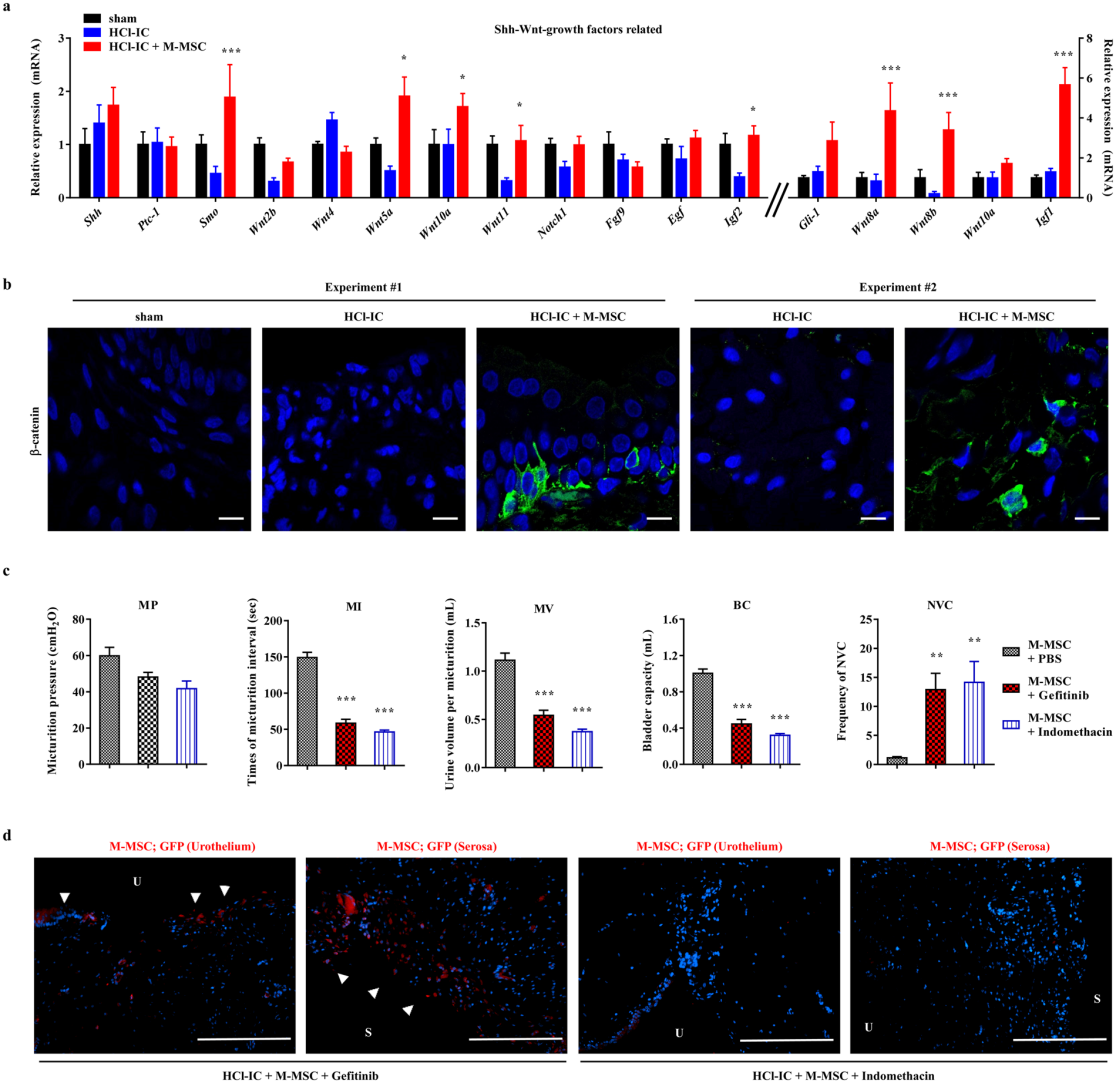
Central role of Wnt and IGF signaling activity on therapeutic effect of M-MSCs. (**a**,**b**) RQ-PCR analyses of expression levels of *Shh*, *Wnt*, and downstream growth factors (**a**) and representative confocal micrographs (magnification ×1,000, scale bar = 10 μm) for immunofluorescence staining of β-catenin protein (**b**) in the bladder tissues of the indicated groups 1 week after the injection of 1 × 10^6^ M-MSCs or PBS vehicle into HCl-IC animals. Expression is presented as % *Gapdh* (n = 10). (**c**) Quantitative data of bladder voiding parameters in rats (from 8 independent animals per group) at 1 week after the injection of 1 × 10^6^ M-MSCs into HCl-IC animals in the absence or presence of indomethacin (Wnt blocker) or Gefitinib (IGF-mediated signaling inhibitor) intervention. All data are presented as the mean ± SEM, **p < 0.01, ***p < 0.001 compared to the HCl-IC group with Bonferroni post-test. (**d**) Detection of M-MSCs stably expressing GFP (red) in the indicated rat bladders by immuostaining (magnification ×200, scale bar = 200 μm). Nuclei were stained with DAPI (blue). Arrow heads indicate the engrafted cells. U; Urothelium, S; Serosa.

To investigate the mechanism of action of M-MSCs therapy for IC/BPS, we examined the expression of genes related to sonic hedgehog (Shh), Wnt, and growth factors that are activated by UCB-MSC therapy to promote epithelial regeneration in a HCl-IC rat model^13^. As shown in Fig. 5a, M-MSC therapy significantly upregulated Shh and Wnt family genes (e.g., *Smo*, *Wnt5a, Wnt8a, Wnt8b, Wnt10a*, and *Wnt11*) as well as their downstream growth factors (e.g., *Igf1*, and *Igf2*), which were characteristically downregulated in bladders of the HCl-IC group. Accordingly, the HCl-IC + M-MSC group bladders were characterized by the increased expression and nuclear localization of β-catenin protein, a surrogate marker for Wnt signaling activation (Fig. 5b and Supplementary Fig. 2). Importantly, the recovery of bladder voiding functions was significantly abrogated by daily injection of indomethacin^32^ or Gefitinib^33^, inhibitors for Wnt and IGF-mediated signaling activity, respectively (Fig. 5c and Supplementary Fig. 3). The treatment of Gefitinib had little effect on the engraftment of the administrated M-MSCs at the injection site in serosa of the bladder; however, little integration of injected cells was observed in urothelium (Fig. 5d). Indomethacin significantly impaired the engraftment of administrated M-MSCs. Thus, these results indicate that the Wnt and IGF signaling cascades play a crucial role in the beneficial outcomes of M-MSC in treating IC/BPS bladders.

### Superior therapeutic potency of M-MSCs than adult tissue counterpart

**Figure 6:**
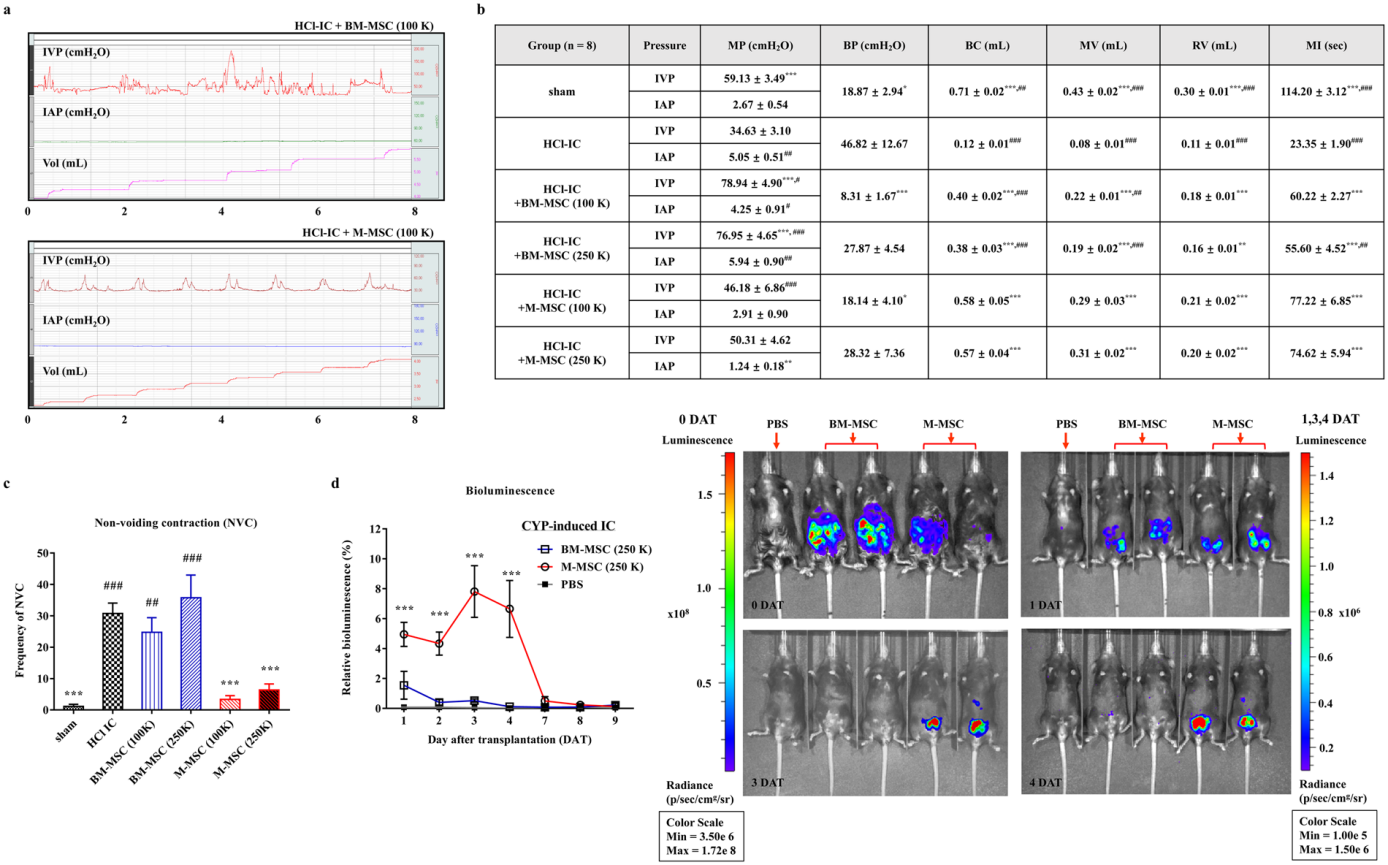
Superior therapeutic efficacy of M-MSCs compared to BM-MSCs. (**a**) Representative awake cystometry results at 1 week after injection of M-MSCs or BM-MSCs at the indicated dosage (K = a thousand) into the bladder of HCl-IC rats. (**b**) The micturition pressure (MP), basal bladder pressure (BP), bladder capacity (BC), micturition volume (MV), residual volume (RV), and micturition interval (MI) were quantified from the voiding pattern analysis. (**c**) Quantification of non-voiding contraction (NVC) in HCl-IC animals transplanted with the indicated number of M-MSCs or BM-MSCs (n = 8). All data are presented as the mean ± SEM from 8 independent animals per group (*p < 0.05, **p < 0.01, ***p < 0.001 compared to the HCl-IC group; ^#^p < 0.05, ^##^p < 0.001, ^###^p < 0.001 compared to M-MSC (250 K) group by one-way analysis of variance with Bonferroni post-test). (**d**) The time course of viability of transplanted M-MSCs or BM-MSCs (2.0 × 10^5^) in cyclophosphamide (CYP)-induced IC/BPS mice. Relative bioluminescent signals for the indicated day after transplantation (DAT) relative to 0 DAT are presented as the mean ± SEM (n = 5; ***p < 0.001 compared to the BM-MSC group). The representative images were obtained at 15 minutes after intraperitoneal injection of 150 μg/ml coelenterazine (200 μL), a substrates for Renilla luciferase. The scale bars for optical image density range at 0 and other day points were presented at left and right sides of image, respectively.

Next, to compare the efficacy of M-MSCs with adult BM-derived MSCs (BM-MSCs), we transplanted the sub-optimal dosage (1.0 × 10^5^ or 2.5 × 10^5^) of cells into the bladder tissues of HCl-IC animals. Importantly, M-MSCs exhibited superior *in vivo* therapeutic potency compared adult tissue counterpart at similar cell doses and exhibited beneficial effects at a dose for which BM-MSCs did not substantially improve aberrant bladder voiding functions such as MV, BC, and MI (Fig. 6a,b, and Supplementary Fig. 4a) in IC/BPS model animals. Unlike BM-MSCs, M-MSC therapy at a low dosage (1 × 10^5^) of M-MSCs remarkably ameliorated the NVC which usually represents urinary urgency symptom in clinical setting (Fig. 6c).

To gain mechanistic insight into the enhanced efficacy of M-MSCs, we examined the kinetic of *in vivo* engraftment/retention after transplantation of the engineered M-MSCs or BM-MSCs which stably express Nano-lantern, a chimera of enhanced Renilla luciferase and Venus fluorescent protein with high efficiency of bioluminescence resonance energy transfer (BRET)^34^. To optimally monitor the bioluminescence signals of transplanted cells, we employed a murine model in which IC/BPS was induced by CYP^35^ and 2 × 10^5^ Nano-lantern^+^ M-MSCs or BM-MSCs were locally injected into bladder tissues. In optical imaging system, bioluminescence intensity from the transplanted M-MSCs were observed until 8 day after transplantation (DAT) (Supplementary Fig. 5a). After rapid decrease (~5% as compared with that when cells were injected) in 1 DAT, the bioluminescence activity from the engrafted M-MSCs sustained until 4 DAT and largely decreasing after 7 DAT (Fig. 6d). Compared to animals treated with BM-MSCs, animals transplanted with M-MSCs demonstrated significantly brighter bioluminescence throughout the whole experimental period (Fig. 6d), indicating the superior engraftment capacity of M-MSCs.

We next monitored the tumorigenicity of transplanted M-MSCs non-invasively by employing longitudinal micro-positron emission tomography/magnetic resonance imaging (μ-PET/MRI) imaging for 12-months after injection. As shown in Supplementary Fig. 5b, only background signal for uptake of 2-[^18^F]-fluoro-2-deoxyglucose (FDG) was detected, and no other [^18^F]-FDG uptake characteristics of tumors were observed in any HCl-IC animal injected with 1 × 10^6^ M-MSCs or PBS vehicle. We confirmed this low tumorigenic potency of M-MSCs by a thorough microscopic investigation of organs in a double-blind necropsy followed by final nanoScanPET/MRI analysis. Taken together, these results suggest that M-MSC-based therapy, with no adverse or safety issues, exerts superior therapeutic and engraftment capacities for treating IC/BPS bladder dysfunction by supporting Wnt signaling-related epithelial regeneration.

### *In vivo* cellular properties of infused M-MSC

**Figure 7:**
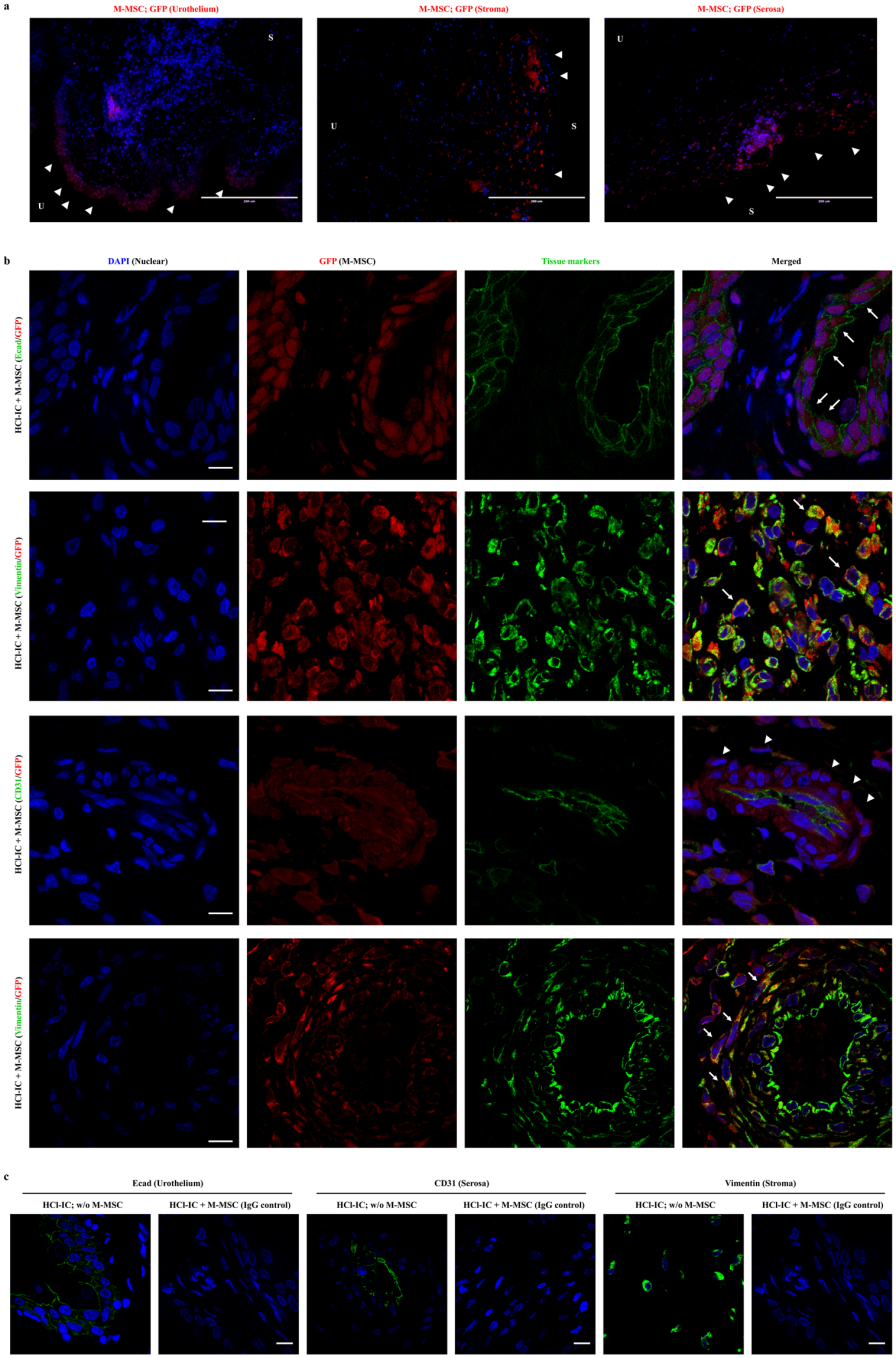
Immunostaining analysis for cellular properties of transplanted M-MSCs. (**a**) Detection of M-MSCs stably expressing GFP (red) in HCl-IC rat bladders at 7 DAT by immuostaining (magnification ×200, scale bar = 200 μm). (**b**) Representative confocal micrographs of bladder sections transplanted with GFP-MSC (HCl-IC + M-MSC) after double staining for GFP^+^ cells (red) and for E-cadherin (Ecad)^+^ urothelial cells, vimentin^+^ stromal and perivascular cells, CD31^+^ endothelial cells (green) (magnification ×1,000, scale bar = 10 μm). Nuclei were stained with DAPI (blue). Arrow heads and arrows indicate the engrafted and differentiated cells, respectively. U; Urothelium, S; Serosa. (**c**) For negative control experiments, co-staining of bladders tissues in HCl-IC + M-MSC group animals with mouse and rabbit IgG control antibodies or co-staining of the indicated tissue markers (green) and GFP (red) in bladder tissues not-injected with GFP + M-MSCs (HCl-IC; w/o M-MSC) for corresponding tissue markers were included (magnification ×1,000, scale bar = 10 μm).

In immunofluorescent analysis of M-MSCs stably expressing green fluorescence protein (GFP), the majority of GFP^+^ cells in HCl-IC animals on 7 DAT were localized at the injection site between muscle and serosa of the bladder and some GFP^+^ cells were observed in the lamina propria and urothelium, but few were detected in the muscular layer (Fig. 7a). In contrast, the bladder tissues of rats injected with GFP^+^ BM-MSCs showed few engrafted GFP^+^ cells (Supplementary Fig. 4b), in line with the bioluminescence assay results (Fig. 6d). To analyze the phenotype of engrafted M-MSCs, multichannel laser scanning confocal microscopy was performed by labeling for engrafted cells (GFP), epithelial cells in urothelium (E-cadherin), stromal cells (vimentin), and endothelial cells (CD31). The GFP^+^ cells found in urothelium expressed the E-cadherin in membrane, indicating their differentiation into epithelial cells (Fig. 7b and c). Majority of GFP^+^ cells found in lamina propria was dispersed as stromal cells with strong co-staining with vimentin protein. Particularly, some GFP^+^ cells in serosa and underneath urothelium was distributed into vessel-like structures (Fig. 7a). Confocal microscopy data indicated that GFP^+^ cells formed in vessel-like clusters little expressed CD31 endothelial cell surface protein, but they were in close contact with the CD31^+^ cells and some of them expressed the vimentin, a pericyte intermediate filament (Fig. 7b). The morphology, localization, and expression of tissue markers indicated that the engrafted M-MSCs hardly differentiate into endothelial cells, but they behaved as pericytes. This perivascular M-MSC phenotype is line with a preceding *in vitro* data that M-MSCs expressed surface antigens for pericyte (PDGFRB, CD146, and NG2) but not endothelial cells (Fig. 1c). These results demonstrate that a considerable amount of transplanted M-MSCs survive and differentiate into multiple cell types in the bladder tissue.

### Longitudinal confocal imaging of infused M-MSCs in living animals

**Figure 8:**
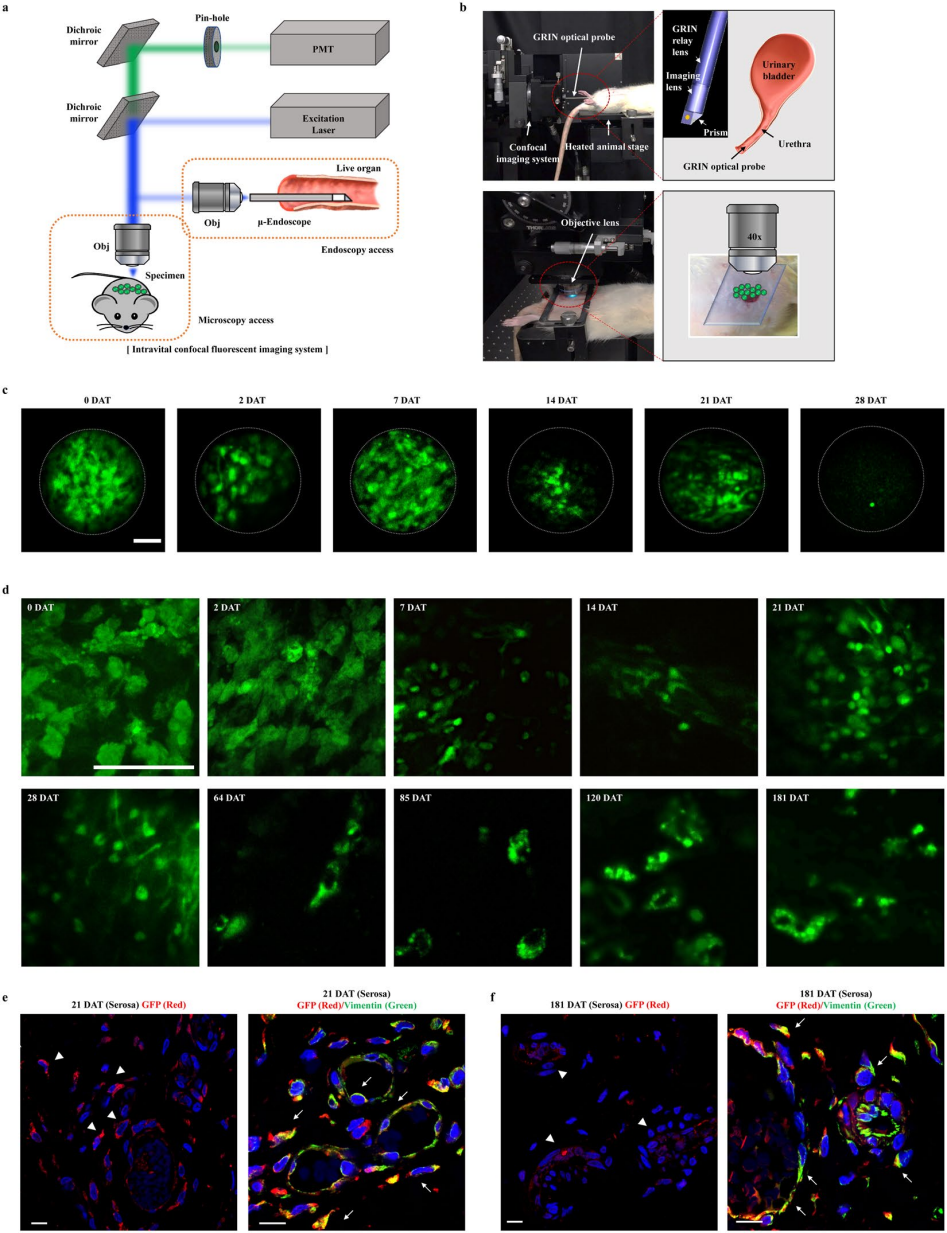
Longitudinal confocal imaging of transplanted M-MSCs in living animals. (**a**,**b**) Schematic overview (**a**) and working station (**b**) of intravital fluorescence imaging in living animal. Front-view GRIN optical probe is endoscopically inserted into the bladder of an anesthetized rat held on an XYZ stage to access the urothelium surface (**b**; upper panel). Objective lens viewed the outer layer of bladder after making a minimal incision in the overlying abodomen (**b**; lower panel). (**c**,**d**) Time-lapse imaging (n = 8) of the engrafted M-MSCs obtained by a GRIN optical endoscopic probe (**c**, magnification ×40) or objective lens (**d**, magnification ×100) in the bladders of HCl-IC rats from 0 day (2 hours) after transplantation (DAT) to 6 months (181 DAT). A majority of transplanted M-MSCs (with GFP fluorescence) were observed in vascular-like structures in the outer layers of the bladder from about 64 DAT. Scale bar = 50 μm. Representative video-clips acquired by these intravital endoscopes and microscopes are available as Supplementary Movie. (**e**) Representative confocal images for integration of transplanted GFP^+^ cells (red) (magnification ×630) and their differentiation into vimentin^+^ cells near CD31^+^ vessels (green) (magnification ×1,000). Arrow heads and arrows indicate the engrafted and differentiated cells, respectively. Nuclei were stained with DAPI (blue). Scale bar = 10 μm.

To investigate *in vivo* properties of engrafted M-MSCs at the cellular level in living animals (Fig. 8a), we employed intravital fluorescence microscopy which enables the study of *in vivo* cellular processes such as cell trafficking, intercellular interactions, and vascular changes^36^. Using front-view endoscopic optical probes directed toward the urothelium (Fig. 8b), we performed longitudinal imaging of infused GFP^+^ M-MSC fluorescence for 6-months. Consistent with optical imaging results, strong focal fluorescence was detected immediately after transplantation by endoscopy. Fluorescence intensity was greatly reduced by 2 DAT but was relatively stable until 21 DAT (Fig. 8c and Supplementary Movie S1). During this period, GFP fluorescence was observed within multiple cellular structures. However, it became weak and blurred beyond 28 DAT.

We next performed high resolution *in vivo* confocal microscopy using objective lenses focused on the outer layer of the bladder through minimal incisions on the abdominal surface (Fig. 8b). Fluorescence intensity gradually decreased until 21 DAT. However, GFP^+^ cells with clear morphology were still observed at 28 DAT and emission was sustained until 181 DAT, whole our observation period (Fig. 8d, Supplementary Movies S2 and S3). Little auto-fluorescence was observed in animals injected with vehicle (Supplementary Fig. 6a and b). In M-MSC-injected animals, GFP^+^ cells with distinct shapes were detected at 28 DAT with broad distribution over the entire bladder (Fig. 8d and Supplementary Fig. 6c). By 2-months, the engrafted cells formed discrete foci and the majority were detected as components of blood-vessel like structures. As aforementioned intravital imaging data, confocal microscopic analysis of bladder tissues indicated that the engrafted M-MSCs functionally integrated into the epithelial cells (E-cadherin^+^) in urothelium (Supplementary Fig. 6d and e) and perivascular cells (vimentin^+^ in close proximity to CD31^+^ cells) at 21 DAT (Fig. 8e) and the pericyte phenotype of M-MSCs remained stable until 6-months (Fig. 8f). Thus, these *in vivo* imaging data support that engrafted M-MSCs may initially replenish the urothelial layer and progressively contribute to perivascular cells.

## Discussion

Although several promising preclinical studies using MSCs for treating IC/BPS have been reported, limited proliferation and impaired stemness during *ex vivo* expansion as well as a general lack of information on the *in vivo* properties of transplanted cells have provoked skepticism over current MSC-based therapy. To overcome these limitations, we demonstrate that hESCs can provide a virtually unlimited source of MSCs and that injection of these hESC-derived M-MSCs results in improved therapeutic outcome for treating IC/BPS in a model animal compared to adult tissue-derived cells, without any adverse safety issues. Furthermore, by longitudinal intravital confocal imaging in living animals, we provide precise information on the *in vivo* distribution and cellular properties of transplanted cells.

IC/BPS is regarded as a heterogeneous multifactorial disease with unclear pathogenesis^37^. The disease etiology is not yet fully understood, and definitive treatments have not been reported. Even though several treatment approaches including several oral medications, bladder instillation therapies, fulguration for Hunner lesion, and hydrodistention have been used, their outcomes have not been satisfactory. IC/BPS may be chronic inflammatory and fibrotic conditions in which mast cell activation, sensory nerve hyperactivation, nitric oxide or autoimmune mechanisms, and glycosaminoglycan layer defects broadly affect the entire bladder, implying that multi-modal treatment approaches could be required^38–40^. For this purpose, SC therapy has several advantages, and indeed MSC-based therapy has proven beneficial in preclinical studies by regenerating damaged tissues through differentiation into target cells and creating a micro-environment favorable to tissue repair^12–14^.

Of note, almost 40% of patients diagnosed with inflammatory bowel syndrome presented with bladder pain, while as many as 40% diagnosed with IC/BPS had symptoms that fulfill the criteria for inflammatory bowel syndrome^29, 30^. Visceral organ communication and the resulting hypersensitivity may be due to the convergence of sensory neural pathways in the dorsal root ganglion, spinal cord, and brain^41^. Other evidence showed sensitization of afferent neurons at the dorsal root ganglion as well as lumbosacral neurons that become hyper-excitable following colonic inflammation^31^. Thus, the activation of afferent nerves in response to mucosal damage may be the result of increased epithelial permeability, allowing electrolytes to have direct access to the visceral sensory neurons. In this regard, the structural regeneration and attenuation of inflammation by M-MSC therapy could prevent visceral organ crosstalk. Accordingly, we found that a single administration of M-MSCs significantly moderated the anatomical interaction between mast cells and nerve fibers, a most convincing parameter in visceral hypersensitivity (Fig. 4a and b), and also the increased expression of Ngf, which is abundantly synthesized and released by both mast cells and nerve fibers (Fig. 4c). Thus, examination of inflammation status in the intestines or pain assessment after M-MSC therapy should be investigated further.

Consistent with previous reports using UCB-MSCs^13^, a single administration of hESC-derived M-MSCs had therapeutic effects in HCl-induced IC animal models (Fig. 2). The engrafted cells were localized mainly between muscle and serosa and more sparsely in lamina propria as vimentin^+^ stromal cells (Fig. 7a,b, and Fig. 8g), which could stimulate Wnt-related epithelial regeneration capacity (Fig. 5). Of importance, engrafted M-MSCs exerted beneficial outcomes even with 10-fold fewer injected cells compared to BM-MSC treatment of the HCl-IC animal model (Fig. 6). This superior potency could be attributed to enhanced *in vivo* engraftment and survival (Fig. 6d), which are critical challenges for therapies based on MSCs from adult tissues^42^. In one report, most (≥99%) intravenously injected MSCs adhered to the lungs, and a mere 2–3% were released into the circulation^43^. As similar, around 5% of locally injected M-MSCs were detected in the bladder tissue in 1 DAT (Fig. 6d). However, our intravital imaging analysis indicated that some M-MSCs were stably engrafted into damaged bladder at the injection site and survived at least 6-months (Fig. 8d and Supplementary Fig. 6c). As a result, the engrafted GFP^+^ cells in bladders of IC/BPS animals were frequently detected as E-cadherin^+^ urothelium as well as vimentin^+^ stromal cells or pericytes (Fig. 8e and Supplementary Fig. 6d). The stable integration of transplanted cells as naive urothelium, stromal, and perivascular cells supporting proper angiogenesis may maximize therapeutic potential by protecting the bladder from urine leakage and by boosting the pro-regenerative microenvironment, respectively. Furthermore, the unlimited proliferation capacity of hESCs could provide voluminous high-quality therapeutic cells with well controlled *in vitro* differentiation characteristics to enhance *in vivo* survival, engraftment, and functionality^23^.

Despite these advantages, one major obstacle to the therapeutic application of hESC-derivatives is the risk of tumorigenesis. However, recent clinical success on eye disorders could allay this general concern of hESC-based therapeutics^44, 45^. Likewise, tumors and abnormal growth of transplanted M-MSCs were not detected by long-term longitudinal nanoScanPET/MRI monitoring and thorough necropsy (Supplementary Fig. 5b). Furthermore, we did not observe any signs of immune rejection and tissue inflammation during the experimental period. This low immunogenic potential of hESCs and differentiated derivatives^46, 47^ may stem from the absence of immunologically relevant cell surface markers, including HLA-DR (Fig. 1c) and costimulatory molecules (CD40, CD40L, B7-1, and B7-2)^24^, which may enable M-MSCs to escape the immune response and to long-term engrafted as perivascular cells. Taken together, the present preclinical data demonstrate that hESC-derived M-MSCs may overcome the limitations of current MSC therapy without adverse outcomes from hESC-derivatives. Recently, several priming strategies have been developed to enforce the function of MSCs derived from adult tissues^48–50^. Thus, the therapeutic efficacy and expansion of M-MSCs should be carefully compared with those of primed MSCs, which could be easily accessible and expandable, and thus seem to be relevant for clinical application.

Another significant hurdle for clinical SC therapy is the paucity of direct long-term analyses of the *in vivo* distribution, phenotype, and functional integration of engrafted cells in injured target organs. Critical functional properties of engrafted SCs include transcriptional activity, external signal transduction, and differentiation potency, all of which are dynamically altered by the disease environment, thereby affecting therapeutic outcome^51^. Thus, longitudinal *in situ* analysis of engrafted cells in living animals could advance our understanding of the cellular mechanisms underlying functional improvement, better evaluate the risks of tumorigenesis and other adverse events after transplantation, and help in the development of optimal treatment protocols. In turn, such data may accelerate the successful translation of these preclinical results to clinical trials.

In the present study, we longitudinally monitored cellular processes of transplanted M-MSCs for 6 months in living animals (Fig. 8). With high resolution from objective lenses, we repeatedly visualized a variety of *in vivo* cellular-level processes (Fig. 8d). However, the large sizes of these objective lenses restricted application mostly to superficial tissues such as skin and surgically exposed internal surfaces. To overcome this drawback, we also employed endo-microscopy with a small-diameter graded-index (GRIN) lens probe to visualize intact tissue in a non-invasive manner (Fig. 8b). Strikingly, we observed similar patterns of fluorescence signals using both approaches. However, the endoscopic approach was able to track the engrafted cells only within 28 DAT (Fig. 8c), while they were visualized under objective lens even at 181 DAT (Fig. 8d). This discrepancy in detection period may be attributed to the limited optical penetration depth of GRIN probes, which is only about 100 μm in most soft tissues^36^. It should be noted that until 1 month post-injection, the majority of engrafted cells were broadly observed on the bladder surface with multiple cellular morphologies, but were focally distributed by 2 months after transplantation (Fig. 8d). Thus, it could be speculated that engrafted M-MSCs may initially replenish the urothelial layer and progressively contribute to establish a micro-environment favorable to tissue repair. For further mechanistic insight, multi-colored lineage tracing with tissue-specific promoters is required.

As SC research in urology is rapidly advancing, successful clinical application of SC therapy is expected in the near future^12, 52, 53^. To successfully translate promising pre-clinical studies into clinical practice, we suggest that hESC-derived M-MSCs are an ideal cost-effective source of therapeutic cells with improved functional potency and minimal tumorigenic and immunogenic capacities. To our knowledge, this is the first study to longitudinally characterize the *in vivo* properties of transplanted SCs at the cellular level in living animals. This innovative approach could advance our understanding of the therapeutic mechanism of current SC therapy.

## Methods

### Study Approval

All animal experiments were approved and performed in accordance with the guidelines and regulations of the Institutional Animal Care and Use Committee of the University of Ulsan College of Medicine (IACUC-2014-14-167).

### Study Design

The purpose of this study was not only to determine greater therapeutic efficacy of human ESC-derived MSCs (M-MSCs) than BM-derived counterparts for treating IC/BPS in a rodent model but also to longitudinally monitor *in vivo* cellular properties of the transplanted cells. *In vitro*, M-MSCs was characterized using morphological and karyotypic analysis, multi-potency, angiogenic potency, and expression of surface markers and stem cell genes. *In vivo*, M-MSCs were administrated into rat injured bladders and the effect on bladder voiding function, urothelium denudation, mast cell infiltration, tissue fibrosis, apoptosis, and tumorigenesis was assessed. Intravital confocal fluorescence imaging in living animals tracked the infused M-MSCs for 6-months after transplantation. For every experimental setting, two independent sets with five independent animals per group were performed. They were randomly allocated to treating groups, the order of injury, the order of cell transplantation or vehicle injection, and the order of cystometry. Information for type and dosage of the injected cells was masked to investigators who were involved in surgical procedures. All cystometric, histological, and gene expression assessment were carried out with investigators who were blinded to treatment groups. Any animals that died unexpectedly by bladder insults or catheter implantation were excluded from any analyses.

### Differentiation and culture of hESC-derived M-MSCs and human BM-derived MSCs

Maintenance of undifferentiated H9-hESCs and differentiation into M-MSCs (Fig. 1a) were performed as previously described^23, 24^. The established M-MSCs were cultured with EGM2-MV medium (Lonza, San Diego, CA, USA) on plates coated with rat tail collagen type I (Sigma-Aldrich, St. Louis, MO, USA) in a humidified atmosphere with 5% CO_2_ at 37 °C. All M-MSCs used in experiments were expanded less than ten passages to ensure multipotency. Characterization of basic features such as surface protein expression, cell proliferation, multipotency (*in vitro* differentiation into osteogenic, chondrogenic, or adipogenic lineages), *in vitro* angiogenesis assays, and karyotyping were performed as previously described^23, 24^. The M-MSC line stably expressing GFP was established by infection of GFP-expressing lentivirus produced as previously described^14^. Human BM-MSCs purchased from Lonza (Basel, Switzerland) were cultured following the manufacturer’s instructions. Cells expanded for fewer than seven passages were used for experiments to ensure multipotency. All cells were tested for mycoplasm content each month (Mycoplasma Hoechst Stain Kit; 3030000; MP Biomedicals, LLC, Santa Ana, CA, USA).

### Animal models and transplantation of M-MSCs

A HCl instillation IC/BPS rat model was established as described previously^13^. One week after the HCl injury, a low abdominal incision was made and the indicated dose of hESC-derived M-MSCs or PBS vehicle was directly injected into the outer layer of the anterior wall and dome of the bladder using a 500 μm syringe and a 26-gauge needle as previously reported^13, 14, 54^. Starting from 1 day before stem cell injection, indomethacin (PMG Pharm Co., Ltd. Ansan, Korea; every 12 h at 2.5 mg/kg) or Gefitinib (Santa Cruz Biotechnology, Santa Cruz, CA, USA; every day at 5 mg/kg) were subcutaneously injected to block Wnt or IGF-mediated signaling, respectively.

### Unanesthetized and unrestrained cystometrogram acquisition (awake cystometry)

Cystometrograms were performed on unanesthetized and unrestrained rats in metabolic cages. Simultaneous catheterizations for intravesical pressure (IVP) and intra-abdominal pressure (IAP) recordings were performed 3 days prior to cystometrogram as described previously^55, 56^. Briefly, following the induction of anesthesia, a polyethylene catheter (PE-50; Becton-Dickinson, Parsippany, NJ, USA) with a cuff was implanted into the dome of the bladder through an abdominal incision. To record IAP, an abdominal balloon (Latex; Daewoo Medical, Incheon, Korea) around the cuff of a catheter tip was placed proximal to the bladder and tied to another catheter with silk thread. A polyethylene catheter (PE-50) was heated in warm water, elongated ~1.5 times its original length at the tip of the inserting side and filled with heparinized saline (100 IU/mL). As the bladder catheter was implanted, the elongated catheter was inserted into the femoral vein. These catheters were then tunneled through the subcutaneous space, exited through the back of the animal and anchored to the skin of the back. Following surgery, each rat was housed individually and maintained in the same manner.

For awake cystometric analysis, the indwelling catheter to the bladder was connected to a two-way valve connected via a T-tube to a pressure transducer (Research Grade Blood Pressure Transducer; Harvard Apparatus, Holliston, MA, USA) and a microinjection pump (PHD22/2000 pump; Harvard Apparatus). Another indwelling catheter connected to a fluid-filled abdominal balloon was connected to another pressure transducer to record the IAP. The micturition volumes were recorded continuously by means of a fluid collector connected to a force displacement transducer (Research Grade Isometric Transducer; Harvard Apparatus) as sterile saline was infused into the bladder at a rate of 0.4 mL/min. The IVP, IAP, and micturition volumes were recorded continuously using an MP150 data acquisition system with Acq Knowledge 3.8.1 software (Biopac Systems, Goleta, CA, USA) at a sampling rate of 50 Hz. The values from all reproducible micturition cycles measured for 8 min from individual animals were used for evaluation.

A non-voiding contraction (NVC) was counted when the increments of IVP exceeded 15 cmH_2_O from baseline without expelled urine. BP was defined as the lowest bladder pressure during filling, MP as the maximum bladder pressure during the micturition cycle, MV as the urine volume of expelled urine, and RV as the urine volume remaining following voiding. BC was defined as MV + RV and MI as the interval between micturition contractions.

### Histological and gene expression analyses

Epithelial denudation was assessed by immunostaining for cytokeratin, mast-cell infiltration by Toluidine blue staining (8544-4125; Daejung Chemicals & Metals, Seoul, Korea), tissue fibrosis by Masson’s trichrome staining (Junsei Chemical, Tokyo, Japan), and apoptosis by TUNEL staining (1 684 795; Roche, Mannheim, Germany) as previously described^14^. Tracking of injected GFP^+^ M-MSCs in the bladder was performed by immunofluorescent staining with a specific rabbit GFP polyclonal antibody (ab290; Abcam, Cambridge, MA, USA). The epithelial, stromal, and endothelial characteristics of GFP^+^ cells were further examined by staining with antibodies against E-cadherin (612130; Clone 36; FITC-conjugated, BD Biosciences, San Diego, CA, USA), vimentin (sc-6260; Santa Cruz Biotechnology), and CD31 (sc-376764; Santa Cruz Biotechnology) respectively. Wnt activation was examined by immunofluorescence staining of β-catenin (sc-7199; Santa Cruz Biotechnology). Immunostaining was visualized using Alexa 488 (A11001)- or 546 (A11010)- conjugated anti-mouse or -rabbit antibodies (Molecular Probes, Grand Island, NY, USA). The nuclei were counterstained with 4′,6-diamino-2-phenylindole (D9542; DAPI, Sigma-Aldrich). Quantitative digital image analysis was performed from three randomly chosen representative areas selected from each slide using Image Pro 5.0 software (Media-Cybernetics, Rockville, MD, USA).

For gene expression analysis, preparation of total RNA was performed using an RNeasy Mini Kit (Qiagen Inc., Valencia, CA), reverse transcription using TaqMan Reverse Transcription Reagents (Applied Biosystems), and real-time quantitative PCR (RQ-PCR) of the indicated transcripts with the PikoReal Real-Time PCR System (Thermo Scientific) and iQ SYBR Green PCR Master Mix (Bio-Rad, Hercules, CA) as described^57^. Three randomly chosen areas from each slide (n = 15) using five independent animals per treatment group was used to quantify the digital image. Similarly, gene expression data are from duplicate RQ-PCR assays (n = 10) from randomly selected five animals per group.

### Animal μ-PET/MRI imaging

Ten HCl-based IC/BPS rats were randomly divided into two groups and injected with 1 × 10^6^ M-MSC (n = 5) or PBS vehicle (n = 5). At 6, 9, and 12 months after injection, μ-MRI/PET imaging was performed using the nanoScanPET/MRI imaging system (1 T, MEDISO, Budapest Hungary). Rats were fasted for 8 hours prior to imaging. Rats were administered 19.7 ± 1.1 MBq in 0.2 mL of 2-[^18^F]-FDG via the tail vein while the rat was under anesthesia (2% isoflurane in 100% O_2_ gas) and warmed using heated air. A T1-weighted gradient-echo (GRE) 3D sequence (TR = 25 ms, TE_eff_ = 3, FOV = 64 mm, matrix = 128 × 128) was acquired during the FDG uptake period. Static PET images were acquired over 15 min in a 1-5 coincident in a single field of view with MRI range. Body temperature was maintained by flowing heated air on the animal bed (Multicell, Mediso, Hungary) and a pressure sensitive pad was used for respiratory triggering. PET images were reconstructed using Tera-Tomo 3D in full detector mode with all the corrections on high regularization and 8 iterations.

### BRET imaging of M-MSCs

M-MSCs were infected with retrovirus containing Nano-lantern construct kindly provided by Prof. Takeharu Nagai^34^. Labeled M-MSCs or BM-MSCs (2 × 10^5^ in 100 μL saline) were injected into the bladders of mice in which chronic bladder inflammation was induced by intraperitoneal administration of cyclophosphamide (CYP, 100 mg/kg, Sigma-Aldrich) every two days for one week^35^. Bioluminescence imaging was performed using IVIS Spectrum Pre-clinical *In vivo* Imaging System and Living Imaging 4.4 software (PerkimElmer, Waltham, MA) following manufacturer’s instruction and a previously published protocol^34^.

### Longitudinal *in vivo* confocal imaging using μ-endoscopy and microscopy

M-MSCs infected with GFP-expressing lentivirus (1 × 10^6^) were directly injected into the bladder of HCl-IC rats and confocal imaging of the infused M-MSCs was performed in living animals using μ-endoscopic optical probes or objective lenses during 6 months after transplantation. A small incision (below 5-mm) was made on the overlying abdominal skin and the outer surface of the bladder was slightly exposed to all contact with the objective lens. The micro-endoscope probe was developed using triplet GRIN lenses structured for front-view imaging^36^. The fabricated endoscope was 1.2 mm in diameter and 5.5 cm in length with transverse and lateral resolutions of 1 μm and 11 μm, respectively, sufficient for resolving single cells. The designed probe was mounted on a custom-built confocal microscope system and optically aligned to the system using a precise XYZ translational stage. By operating continuous 488 nm laser excitation, the GFP emission signal was detected and 2D-fluorescent images were acquired at 30 frames/s.

### Statistics

Data are reported as the mean ± standard error of the mean (SEM) and were analyzed by GraphPad Prism 6.0 software (GraphPad Software, La Jolla, CA). Treatment group differences were tested for significance by one-way or two-way ANOVA followed by Bonferroni post hoc tests. A p-value < 0.05 was considered statistically significant.

## Electronic supplementary material

Supplementary Information

Movie S1.

Movie S2.

Movie S3
