# Supplementary material for: Improved efficacy and in vivo cellular properties of human embryonic stem cell derivative in a preclinical model of bladder pain syndrome
Source: Sci Rep. 2017 Aug 21;7:8872. doi: 10.1038/s41598-017-09330-x (PMC5567131; doi:10.1038/s41598-017-09330-x)
Supplement: Supplementary file 1 — Supplementary Information [file 41598_2017_9330_MOESM1_ESM.pdf]

Supplementary Information

Improved efficacy and in vivo cellular properties of human embryonic stem cell derivative in a preclinical model of bladder pain syndrome

Aram Kim[1,11,†], Hwan Yeul Yu[1,2,†], Jisun Lim[2,3,†], Chae-Min Ryu[1,2], YongHwan Kim[2,3], Jinbeom Heo[2,3], Ju-Young Han[1,2], Seungun Lee[2,3], Yoon Sung Bae[4], Jae Young Kim[4], Dong-Jun Bae[5], Sang-Yeob Kim[5], Byeong-Joo Noh[6], Ki-Sung Hong[7], Ji-Yeon Han[8], Sang Wook Lee[9], Miho Song[1], Hyung-Min Chung[7,10], Jun Ki Kim[4,5], Dong-Myung Shin[2,3,*], Myung-Soo Choo[1,*]

[†]These authors contributed equally to this work.

[*]Correspondence:

Dong-Myung Shin, Ph.D., Department of Biomedical Sciences, Asan Medical Center, University of Ulsan College of Medicine, 88 Olympic-ro 43-gil, Songpa-gu, Seoul 05505, Korea

Tel: +82-2-3010-2086; Fax: +82-2-3010-8493; Email: d0shin03@amc.seoul.kr

Myung-Soo Choo, M.D., Ph.D, Department of Urology, Asan Medical Center, University of Ulsan College of Medicine, 88 Olympic-ro 43-gil, Songpa-gu, Seoul 05505, Korea

Tel: +82-2-3010-3735; Fax: +82-2-477-8928; Email: mschoo@amc.seoul.kr

Supplementary Figures and legends

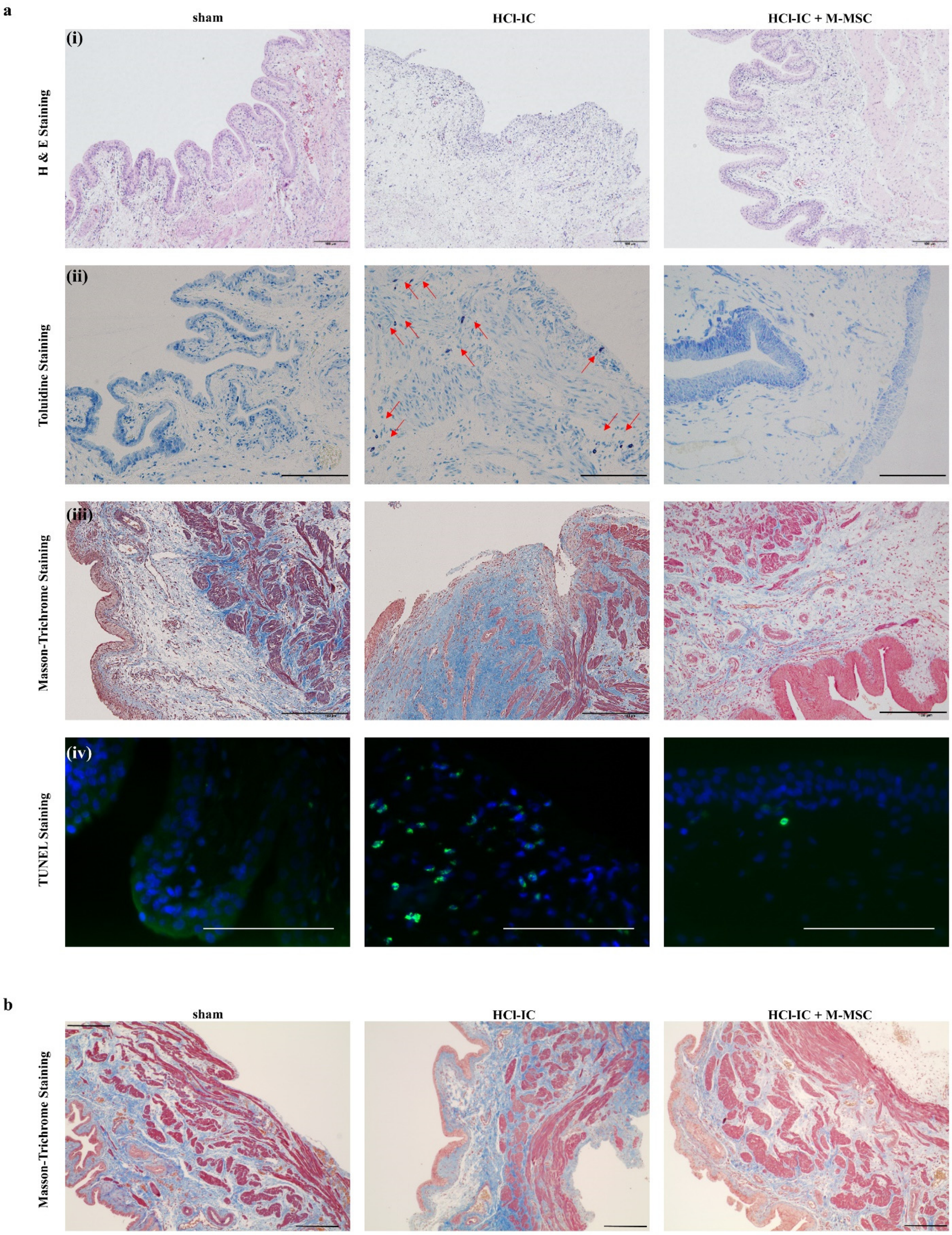

Supplementary Figure 1. The beneficial effect of M-MSCs therapy for treating HCl induced IC.

(a) Histological analysis of M-MSC injection effects on HCl-induced bladder injury. i) Hematoxylin and Eosin (H&E) staining (magnification ×100, scale bar = 100 μm), ii) Toluidine blue staining (magnification ×200, scale bar = 100 μm), iii) Masson's trichrome staining (magnification ×200, scale bar = 100 μm), and iv) TUNEL assay (magnification ×400, scale bar = 100 μm) in the indicated bladder tissues. Nuclei were stained with Mayer's hematoxylin (i, ii, and iii) or DAPI (blue, iv). Arrows (ii) indicate infilated mast cells. **(b)** Little histological alternation of bladder muscles examined by Masson's trichrome staining (magnification ×100, scale bar = 100 μm).

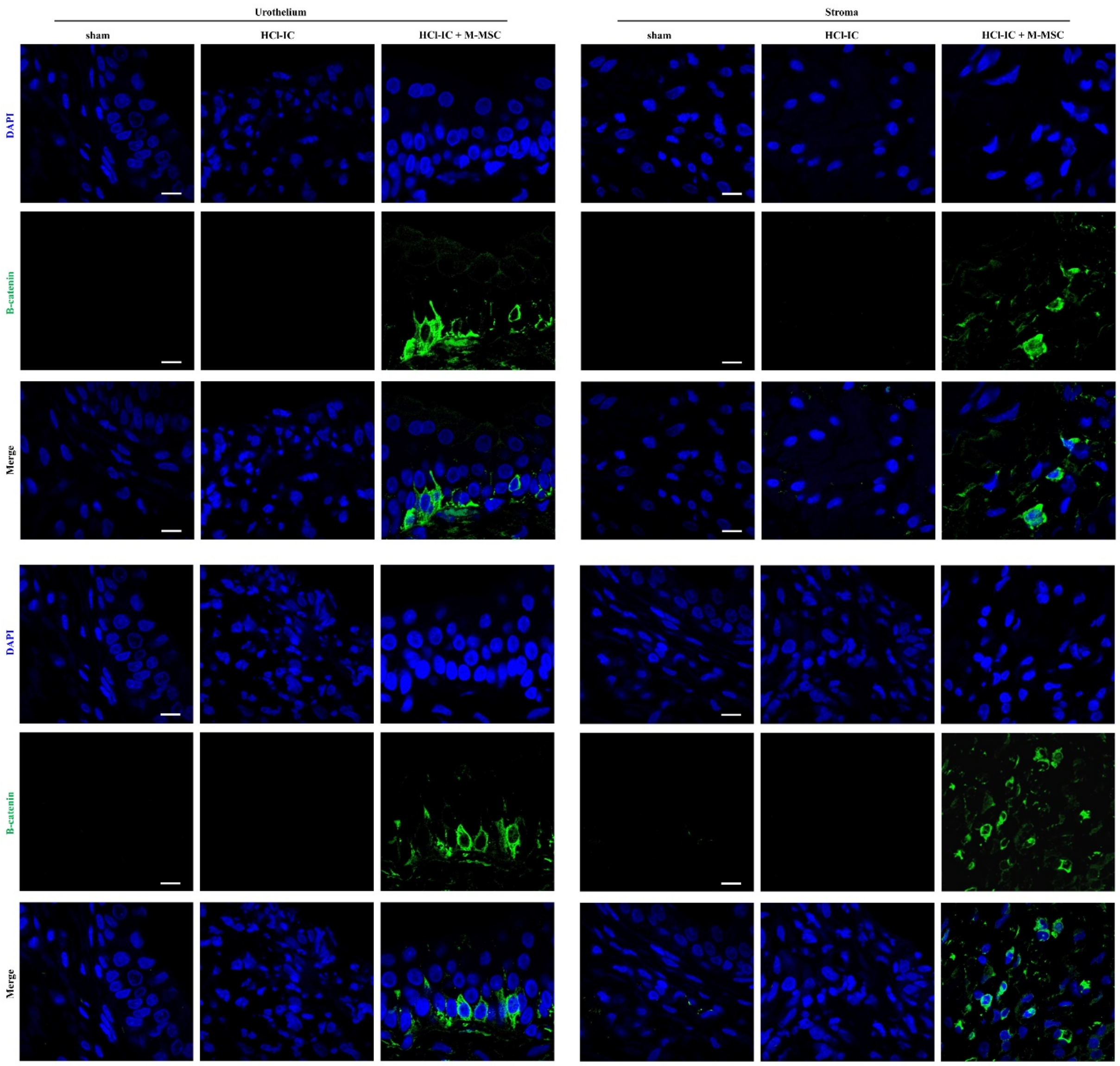

Supplementary Figure 2. Immunostaining of β-catenin in the bladder after M-MSC therapy. Representative confocal micrographs (magnification ×1,000, scale bar = 10 μm) for immunofluorescence staining of β-catenin protein (green) in the urothelium (left panel) and stroma (right panel) sections of bladder tissues at 1 week after the injection of $1{\times}10^6$ M-MSCs or PBS vehicle into HCl-IC animals. Nuclei were stained with DAPI (blue). Sham: sham-operated.

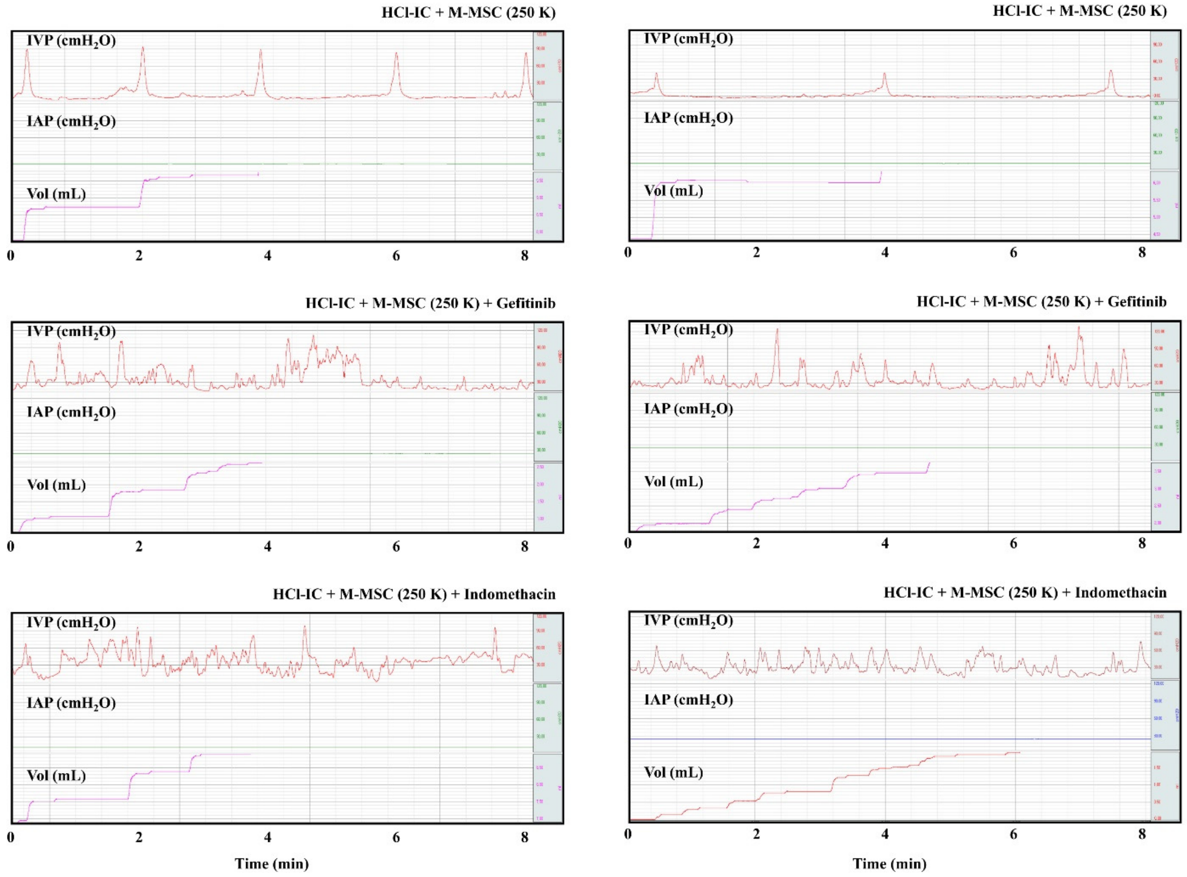

Supplementary Figure 3. Effect of Wnt or IGF signaling inhibition on M-MSCs therapy for treating HCl-induced IC.

Representative awake cystometry results in the absence or presence of indomethacin (Wnt blocker) or Gefitinib (used to inhibit IGF-mediated signaling) at 1 week after the injection of 1×10^6 M-MSCs into HCl-IC animals (8 independent animals per group). IVP; intravesical pressure, IAP; intra-abdominal pressure.

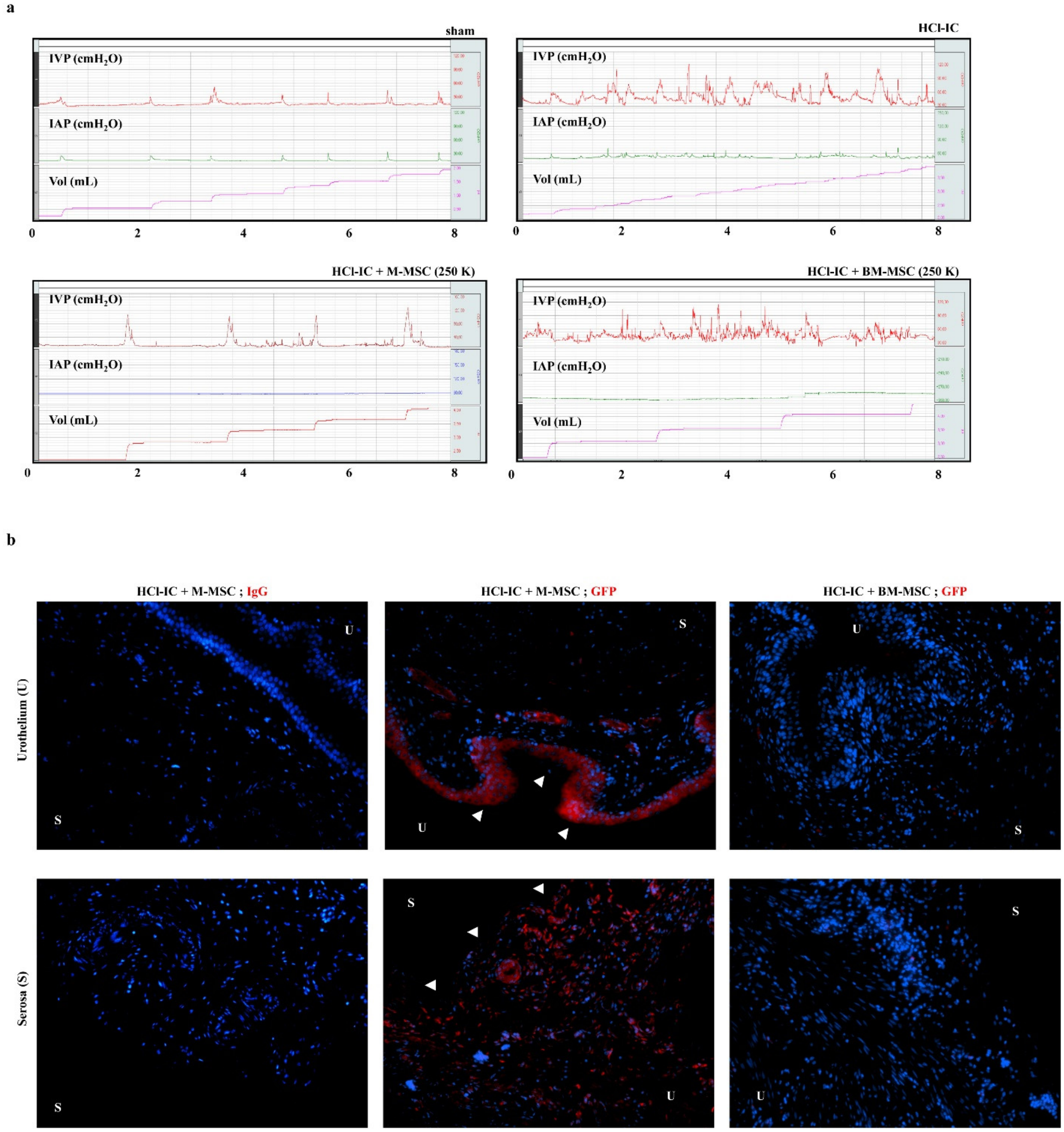

Supplementary Figure 4. Limitation of BM-MSCs about therapeutic efficacy and in vivo engraftment.

(a) Representative awake cystometry results at 1 week after injection of 0.25×10^6 M-MSCs or bone-marrow (BM)-MSCs into the bladder of HCl-IC rats. IVP; intravesical pressure, IAP; intra-abdominal pressure. Sham: sham-operated. K: a thousand. **(b)** Immunofluorescence staining of GFP for detection of the injected GFP^+ BM-MSCs in HCl-IC rat bladders at 7 days after transplantation (magnification × 200). Nuclei were stained with DAPI (blue).

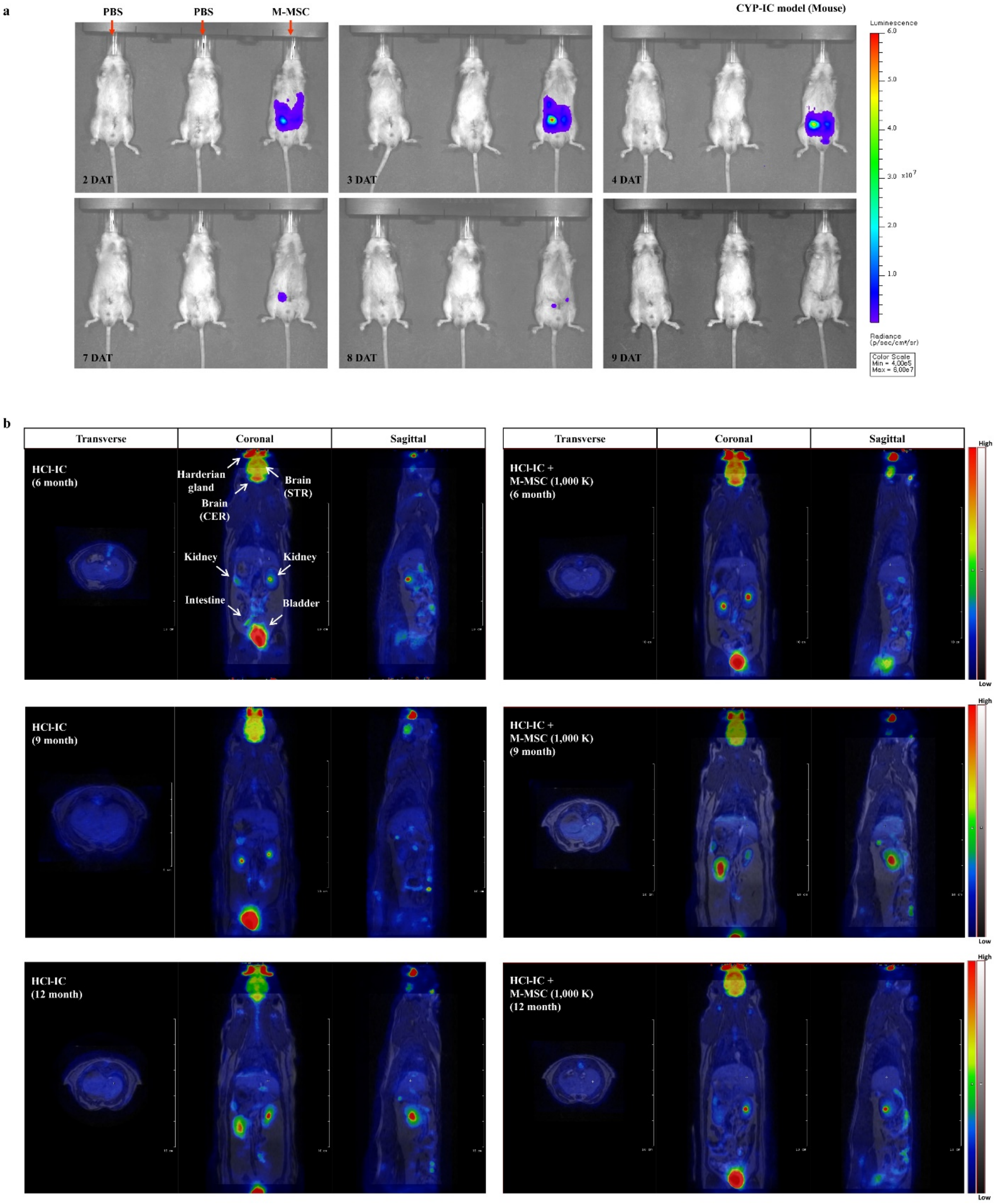
a
PBS
PBS
M-MSC
CYP-IC model (Mouse)
2 DAT
3 DAT
4 DAT
7 DAT
8 DAT
9 DAT
Luminescence
6.0
5.0
4.0
3.0 x10^7
2.0
1.0
Radiance (p/sec/cm²/sr)
Color Scale Min = 4.00e5 Max = 6.00e7
b
Transverse
Coronal
Sagittal
HCl-IC (6 month)
Harderian gland
Brain (STR)
Brain (CER)
Kidney
Kidney
Intestine
Bladder
HCl-IC + M-MSC (1,000 K) (6 month)
HCl-IC (9 month)
HCl-IC + M-MSC (1,000 K) (9 month)
HCl-IC (12 month)
HCl-IC + M-MSC (1,000 K) (12 month)
High
Low

Supplementary Figure 5. Bioluminescence and micro-PET/MRI imaging asssay of transplanted M-MSCs.

(a) Imaging of biolouminescence activies from injected M-MSCs at the indicated day after transplantation (DAT). The representative images were obtained at 15 minutes after intraperitonieal injection of 150 μg/ml coelenterazine (200 μL), a substrates for Renilla luciferase. PBS vehicle or 2×10^5 Nano-lantern expressing M-MSCs or BM-MSCs were administered directly into bladders of a murine model of IC/BPS induced by intraperitoneal administration of cyclophosphamide (100 mg/kg) every two days for one week. **(b)** Longitudinal PET/MRI imaging of transplanted M-MSCs. The transverse, coronal, and satittal views of fused MRI (T1 GRE EX) and PET images (15 min scan after [^{18}F]-FDG injection) of HCl-IC rats at 6, 9, and 12 months after injection of PBS vehicle (left) or transplantation of 1×10^6 M-MSCs (right, HCl-IC + M-MSC) (n=5).

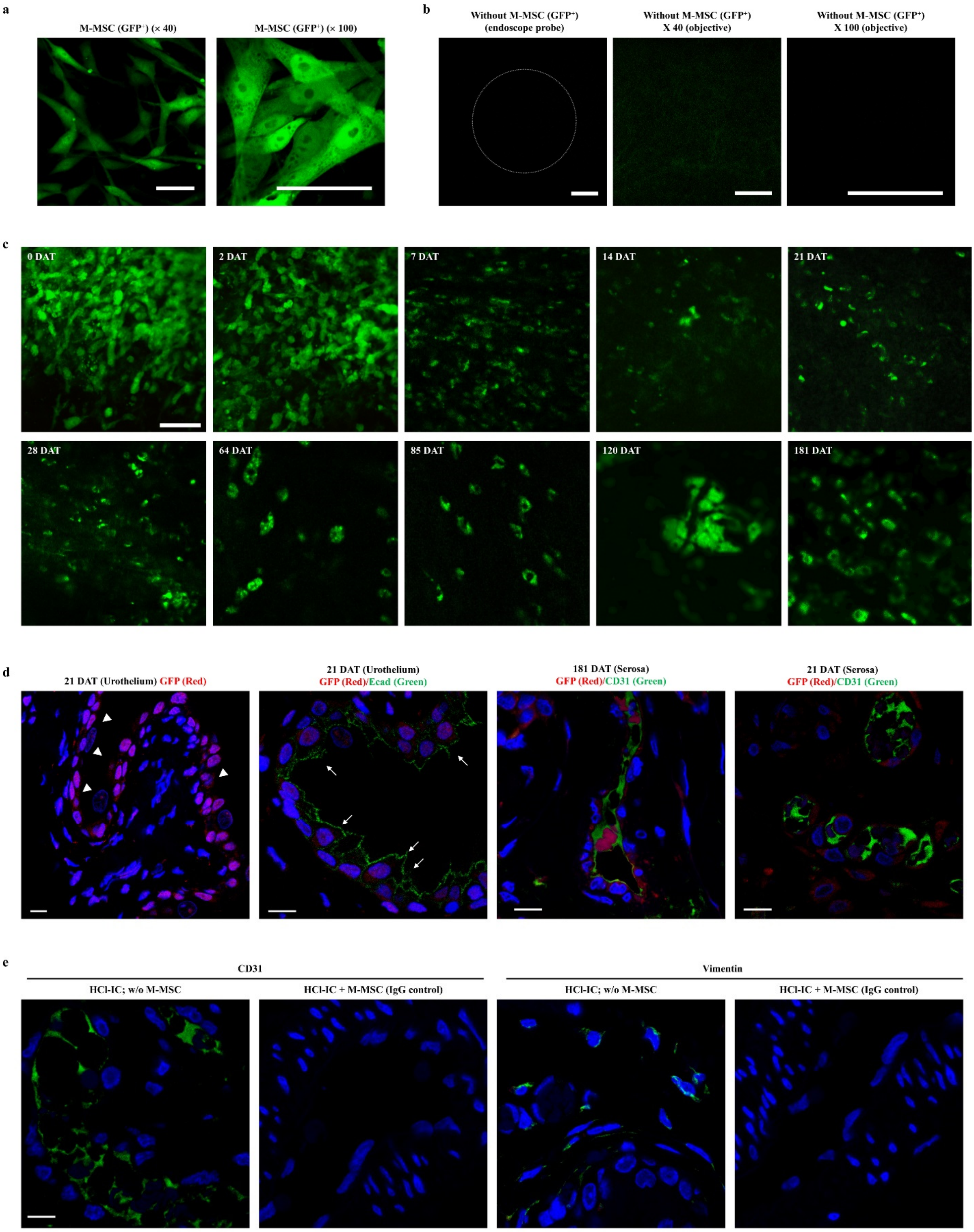

a
M-MSC (GFP+) (× 40)
M-MSC (GFP+) (× 100)
b
Without M-MSC (GFP+) (endoscope probe)
Without M-MSC (GFP+) X 40 (objective)
Without M-MSC (GFP+) X 100 (objective)
c
0 DAT
2 DAT
7 DAT
14 DAT
21 DAT
28 DAT
64 DAT
85 DAT
120 DAT
181 DAT
d
21 DAT (Urothelium) GFP (Red)
21 DAT (Urothelium) GFP (Red)/Ecad (Green)
181 DAT (Serosa) GFP (Red)/CD31 (Green)
21 DAT (Serosa) GFP (Red)/CD31 (Green)
e
CD31
Vimentin
HCl-IC; w/o M-MSC
HCl-IC + M-MSC (IgG control)
HCl-IC; w/o M-MSC
HCl-IC + M-MSC (IgG control)

Supplementary Figure 6. Intravital confocal imaging of the transplanted M-MSCs.

(a) Representative images for detecting GFP fluorescence during cultivation of GFP^+ M-MSCs (magnification ×40 and ×100). **(b)** Only weak fluorescent signals were observed in animals without transplantation of GFP^+ M-MSCs in intravital imaging with an endoscopic probe (left) or objective lens (middle; magnification ×40, right; magnification ×100). **(c)** Longitudinal detection of the engrafted M-MSCs in living animals. Time-lapse images were obtained by objective lens (magnification ×40) over the bladder of HCl-IC rats from 0 day (2 hours) after transplantation (DAT) to 6 months (181 DAT). Objective lene accessed the outer layer of bladder through a minimal incision in the overlying abodomen. Scale bar = 50 μm. Representative videoclips of these intravital microscopic studies are available as **Supplementary Movies**. **(d and e)** Representative confocal microscopic images for integration of transplanted GFP^+ cells (red) (magnification ×630) and their differentiation into E-cadherin $(Ecad)^+$ urothelial or $CD31^+$ endothelial cells (green) (magnification ×1,000) at the indicated DAT **(d)** or for co-staining of bladder tissues in HCl-IC + M-MSC group animals at 181 DAT with mouse and rabbit IgG control antibodies or co-staining of bladder tissues not-injected with GFP+ M-MSCs (HCl-IC; w/o M-MSC) (**e;** magnification ×1,000). Nuclei were stained with DAPI (blue). Arrow heads and arrows indicate the engrafted and differentiated cells, respectively. Nuclei were stained with DAPI (blue). Scale bar=10 μm.

Supplementary Movies and legends

Movie S1. Intravital endoscope imaging at 21 DAT (**Fig. 8c**)

Movie S2. Intravital microscope 120 DAT with 100×magnification (**Fig. 8d**)

Movie S3. Intravital microscope 181 DAT with 40×magnification (**Supplementary Fig. 6c**)

Supplementary Movies at other days after transplantation would be available at website.
